# The Gut–Liver Axis Mediates Liver Injury Induced by Low-Molecular-Weight Polycyclic Aromatic Hydrocarbons Through TLR4-Dependent Inflammation and Bile Acid Dysregulation

**DOI:** 10.3390/foods15193387

**Published:** 2026-09-23

**Authors:** Shiyao Jiang, Jiali Qin, Yunting Li, Xiuxia Dong, Yixuan Hu, Kefan Jiang, Haitao Ma, Zhengyi Zhang, Chengyun Li, Junling Wang

**Affiliations:** 1 Department of Toxicology, School of Public Health, Lanzhou University, Lanzhou 730000, China; qq611943512@163.com (S.J.);; 2 School of Public Health, Southeast University, Nanjing 210009, China; 3 The Second Hospital & Clinical Medical School, Lanzhou University, Lanzhou 730000, China

**Keywords:** LMW-PAHs, dietary contaminants, gut–liver axis, gut microbiota, liver injury

## Abstract

Low-molecular-weight polycyclic aromatic hydrocarbons (LMW-PAHs) are ubiquitous dietary contaminants in grilled, smoked, and thermally processed foods, yet their hepatotoxic mechanisms remain poorly understood. We established a rat model co-exposed to fluorene (Flu) and phenanthrene (Phe) at low, medium, and high doses, validated by urinary metabolites and colonic AHR/CYP1A1. We assessed colonic barrier integrity, gut microbiota (GM) composition, lipopolysaccharide (LPS) translocation, hepatic inflammation, oxidative stress, lipid metabolism, and gut–liver metabolomic profiles. Flu/Phe co-exposure reduced tight junction proteins and altered GM composition—characterized by increased Gram-negative bacteria and reduced gut health indices—facilitating LPS translocation and upregulating the hepatic TLR4/MyD88/NLRP3 signaling axis, which was associated with inflammation, oxidative stress, and lipid metabolic disorders. The medium-dose group exhibited the most pronounced effects, suggesting a possible non-monotonic dose–response relationship. Metabolomic analysis revealed bile secretion as the most significantly enriched pathway common to colon and liver. Mechanistically, combined Flu/Phe exposure disrupted bile acid homeostasis by suppressing FXR/SHP while upregulating CYP7A1 and CYP8B1. Collectively, these findings demonstrate that LMW-PAHs induce liver injury through two distinct mechanisms: GM dysbiosis-driven LPS/TLR4 signaling and FXR/SHP-mediated bile acid dysregulation. The gut–liver axis thus provides candidate molecular nodes for LMW-PAH dietary risk assessment, though validation at dietary-relevant doses is required given the high-dose mechanistic design of this study.

## 1. Introduction

Polycyclic aromatic hydrocarbons (PAHs) are a class of compounds ubiquitous in ambient air, water, and food [1]. They consist of two or more fused benzene rings and are recognized as persistent organic pollutants (POPs) that pose a serious risk to human health [2]. Human exposure occurs through inhalation, dermal contact, and dietary ingestion [3]. Among these routes, dietary intake predominates, because PAHs are generated during common food processing methods including smoking, barbecuing, and deep-frying [4]. Furthermore, airborne PAHs can elevate PAH levels in food through atmospheric deposition, adsorption, and particle attachment [5,6]. Once ingested, PAHs are metabolized primarily in the liver, producing reactive metabolites—including epoxides, catechols, trans-dihydrodiols, and ortho-quinones—that can form adducts with DNA, RNA, and proteins in hepatocytes, thereby disrupting normal hepatocyte function [7,8,9].

The toxicological impact of PAHs on the liver depends not only on the route of absorption but also on the number of benzene rings [10,11]. PAHs with four or more rings are classified as high-molecular-weight PAHs (HMW-PAHs), whereas those with fewer than four rings are considered low-molecular-weight PAHs (LMW-PAHs) [12]. Compared with HMW-PAHs, LMW-PAHs exhibit greater water solubility and enhanced bioavailability [13,14]. Both classes are oxidatively processed by cytochrome P450 enzymes (CYPs), and the metabolism of LMW-PAHs generates water-soluble metabolites that can interact with DNA, forming adducts that provoke gene mutations and cancer [15]. However, research on PAHs in the context of the gut–liver axis has focused almost exclusively on HMW-PAHs. For example, benzo(a)pyrene (BaP) exposure markedly reduces the mRNA expression of zonula occludens-1 (ZO-1) and Occludin in the murine colon, indicating increased colonic permeability and gut injury. BaP exposure is also associated with GM dysbiosis, including proliferation of Gram-negative bacteria, which may promote the production of pro-inflammatory molecules such as lipopolysaccharide (LPS). These molecules can translocate across the compromised gut barrier into the systemic circulation and reach the liver, where they elicit an immune response [16].

The gut–liver axis is a bidirectional pathway linking the intestine and the liver through three essential components: the GM, the portal vein, and the biliary system [17]. Under normal physiological conditions, the liver delivers nutrients into the circulation for distribution to the gut. The intestinal mucosal barrier facilitates nutrient absorption while simultaneously preventing pathogenic bacteria and harmful GM metabolites—including LPS, bacterial DNA, flagellin, and peptidoglycan—from entering the portal vein and reaching the liver [18,19]. When the gut mucosal barrier is compromised, however, these harmful metabolites can enter the portal circulation as antigenic signals and induce hepatic inflammatory responses. Concurrently, the liver reciprocally influences GM composition through the regulation of bile acid metabolism. Recent evidence demonstrates that HMW-PAHs, specifically BaP, reduce beneficial bacteria such as *Lactobacillus* spp. and *Bifidobacterium* spp. while promoting the growth of potentially pathogenic, endotoxin-producing Gram-negative bacteria. A primary consequence of this dysbiosis is the translocation of LPS across the gut barrier into the portal vein and its subsequent transport to the liver, where it amplifies the hepatic inflammatory response. Additionally, BaP exposure substantially alters bile acid production, secretion, and secondary metabolic activity in the gut: the abundance of Clostridia and *Bacteroides*, which facilitate bile acid conversion, declines, diminishing the capacity for bile acid biotransformation. This reduction weakens the inhibitory effect of bile acids on pathogenic bacterial strains, further exacerbating gut dysbiosis [20,21] and leading to pathological accumulation of primary bile acids in the liver, which can directly impair hepatocyte health and promote inflammation and fibrosis [22,23].

Despite this growing understanding of HMW-PAHs, the hepatotoxic mechanisms of LMW-PAHs operating through GM disruption via the gut–liver axis remain largely unexplored. Notably, phenanthrene (Phe) and fluorene (Flu) have been identified as the most abundant LMW-PAH species in urban air monitoring studies across China [24,25]. Because LMW-PAHs are ingested mainly through food, elucidating their hepatotoxic mechanisms directly informs the hazard characterization underlying food safety risk assessment. Here, we examined combined exposure to Phe and Flu in a rat model to evaluate their effects on the liver and colon, with particular emphasis on GM composition, gut barrier integrity, and gut–liver axis metabolomics.

## 2. Materials and Methods

### 2.1. Animal Experimental Procedure

Eight-week-old SPF-grade male Wistar rats (Lanzhou Veterinary Research Institute, Chinese Academy of Agricultural Sciences) were housed under controlled conditions (22 ± 1°C; 55 ± 10% humidity; 12 h light/dark cycle) with ad libitum access to food and water. The experimental protocol was approved by the Ethics Committee of Lanzhou University School of Public Health (IRB23061401).

After one week of acclimatization, rats were randomly assigned to four groups (*n* = 8 per group). Within each group, three rats were fixed for histopathological analysis, and the remaining five rats were used for molecular, biochemical, LPS, microbiome, and metabolomic analyses; this allocation was prespecified in the study design. The experimental design is summarized in Figure 1. The control group (C) received corn oil without supplemental Phe or Flu. The low-dose group (L) received corn oil containing Phe and Flu at 13 mg/kg body weight (b.w.) per day each compound at 13 mg/kg/day (total PAH load: 26 mg/kg/day). The medium-dose group (M) received each compound at 26 mg/kg/day (total: 52 mg/kg/day). The high-dose group (H) received each compound at 130 mg/kg/day (total: 260 mg/kg/day). Doses were selected on the basis of classical toxicological principles, using fractions (1/100, 1/50, and 1/10) of the reported oral LD_50_ of 1300 mg/kg for both Phe and Flu, to elicit reproducible effects while avoiding acute mortality [26]. We acknowledge that these doses substantially exceed estimated human dietary intake (~0.12 μg/kg b.w./day for total PAHs [27]); however, such doses are necessary to elicit detectable pathway responses and construct a full dose–response profile in a mechanistic study. All treatments were administered by oral gavage daily for two months. Prior to sample collection, rats were fasted for 12 h. Urine, feces, blood, liver, and colon contents were collected for subsequent analyses.

### 2.2. Detection of 9-OH Phe and 2-OH Flu in Urine

Urinary 9-OH Phe and 2-OH Flu were quantified by high-performance liquid chromatography (Agilent HPLC-1260, Agilent Technologies, Santa Clara, CA, USA) using a ZORBAX SB-C18 column (5 μm, 150 mm × 4.6 mm, Agilent Technologies, Santa Clara, CA, USA) under isocratic elution at a flow rate of 0.95 mL/min with detection at 280 nm. The mobile phase consisted of methanol/sodium acetate buffer (66:34, *v*/*v*; pH 4.8). The injection volume was 20 μL, and the column temperature was maintained at 30 °C. Sample pretreatment details are provided in Appendix A.

### 2.3. H&E Staining

Colon and liver tissues were fixed in 10% formalin saline for 24 h, embedded in paraffin, sectioned at 5 μm, and stained with hematoxylin and eosin (H&E).

### 2.4. Oil Red O (ORO) Staining

Frozen liver sections (10 μm) were air-dried at room temperature for 5 min to ensure tissue adherence. Oil Red O working solution (150 μL; 5% Oil Red O in 60% isopropanol) was applied to each section and incubated for 10 min. Slides were subsequently washed with distilled water and mounted with 75% glycerol.

### 2.5. Detection of Liver Injury Markers

Alanine aminotransferase (ALT), aspartate aminotransferase (AST), non-esterified fatty acids (NEFA), total cholesterol (T-CHO), and triglycerides (TG) were measured using commercial kits (Nanjing Jiancheng, Nanjing, China) according to the manufacturer’s instructions.

### 2.6. Liver Oxidative Stress Assay

8-Hydroxy-2′-deoxyguanosine (8-OHdG, Shanghai Enzyme-Linked Bio, Shanghai, China) was measured using a kit from Wuhan Eliot. Superoxide dismutase (SOD, Nanjing Jiancheng, Nanjing, China) and malondialdehyde (MDA, Nanjing Jiancheng, Nanjing, China) were measured using kits from Nanjing Jiancheng. All assays followed the manufacturer’s instructions.

### 2.7. Gut Microbiota Analysis

GM composition in rat colon contents was profiled by 16S rRNA gene sequencing. Detailed experimental procedures are provided in Appendix A.

### 2.8. LPS Detection

LPS concentrations in liver, serum, and colon contents were quantified using a Rat LPS ELISA Kit (Shanghai Enzyme-Linked Bio, Shanghai, China).

### 2.9. RT-qPCR Analysis

Total RNA was extracted from colon and liver tissue samples (0.1 g) using RNAiso Plus reagent (TaKaRa, Kyoto, Japan). cDNA was synthesized using the PrimeScript™ RT kit (TaKaRa, Japan) and amplified using TB Green^®^ Premix Ex Taq™ II (Tli RNaseH Plus) (TaKaRa, Japan) on a real-time PCR system (Bio-Rad, Hercules, CA, USA). Primers and PCR cycling conditions are provided in Appendix A.

### 2.10. Western Blotting

Colon and liver tissues were lysed with RIPA lysis buffer (Wuhan BOSTER, Wuhan, China) containing 1% PMSF and 1% phosphatase inhibitor (Wuhan BOSTER). Protein lysates were quantified, separated by electrophoresis, transferred to membranes, blocked, incubated with primary and secondary antibodies, and visualized. Detailed protocols are provided in Appendix A.

### 2.11. Metabolomics Analysis of Liver and Colon Contents

Metabolites from rat liver and colon contents were analyzed on an HPLC-MS platform [28]. Detailed operational procedures are provided in Appendix A.

### 2.12. Statistical Analysis

Statistical analyses were performed using GraphPad Prism 9.0, QIIME2 (microbiome α- and β-diversity), and MetaboAnalyst 5.0 (metabolomics). Normality was assessed using the Shapiro–Wilk test and homogeneity of variance using Levene’s test; all continuous variables satisfied these assumptions. Data were therefore analyzed by one-way ANOVA followed by Tukey’s post hoc test, except for phylum-level relative abundances, which were compared by the Mann–Whitney U test. β-diversity was assessed by PERMANOVA on NMDS ordination. Differential metabolites were identified by Student’s *t*-test with Benjamini–Hochberg false-discovery-rate correction and reported with fold-change and VIP values. KEGG pathway enrichment was assessed by a hypergeometric test, and enriched pathways were visualized with the KEGG Mapper tool. Supervised multivariate models were evaluated by OPLS-DA with permutation testing (200 permutations). Effect sizes and 95% confidence intervals are reported where applicable. A two-sided ***p*** < 0.05 (FDR-adjusted q < 0.05 for metabolomics) was considered statistically significant. An a priori sample-size or power calculation was not performed; the numbers of animals per analysis were determined by the fixed/fresh-tissue allocation described in Section 2.1. All analyses were performed blinded to group allocation.

## 3. Results

### 3.1. Urinary Levels of 2-OH Flu and 9-OH Phe

Following combined Flu and Phe exposure, urinary concentrations of 2-OH Flu and 9-OH Phe increased significantly with increasing dose (***p*** < 0.05; Figure 2), reaching maximum levels of 26.3 μg/mL and 4.6 μg/mL, respectively, providing supporting (semi-quantitative) confirmation that the organism effectively metabolized both compounds.

### 3.2. Effects of Combined Flu/Phe Exposure on AHR and CYP1A1 Expression in Colon and Liver

To investigate whether combined Flu/Phe exposure upregulates CYP1A1 through AHR activation in the colon, we quantified AHR and CYP1A1 mRNA expression by RT-qPCR. Combined Flu/Phe exposure significantly upregulated both transcripts in a dose-dependent manner (***p*** < 0.05; Figure 3A,B). Western blot analysis confirmed that the corresponding protein levels were also significantly increased (***p*** < 0.05; Figure 3D,E), consistent with the gene expression data. We further examined AHR and CYP1A1 expression in the liver. Both the mRNA and protein levels of AHR and CYP1A1 were significantly elevated in liver tissues across all exposure groups (***p*** < 0.05; Figure 3F–J). Together with the urinary metabolite data (Figure 2), the coordinate upregulation of AHR and CYP1A1 in colon and liver confirmed the successful construction of the combined Flu/Phe exposure model.

### 3.3. Histopathological Analysis

H&E staining revealed varying degrees of pathological alteration in the colon across groups (Figure 4A). In the C group, the colonic mucosa was structurally intact, with normal cell morphology in all layers, abundant goblet cells in the lamina propria, and well-defined crypt architecture. In the L group, mild mucosal damage was observed (blue marker). In the M and H groups, severe mucosal damage (blue marker), abnormal cell morphology (red marker), goblet cell loss (green marker), and crypt distortion were evident. These results demonstrate that combined Flu/Phe exposure induces colonic injury.

H&E staining of liver sections (Figure 4B) showed that the C group exhibited intact hepatic architecture with well-defined radial hepatic cords and no evident pathological changes, such as edema or congestion. In the L, M, and H groups, loss of hepatic cord structure, inflammatory cell infiltration, and marked reduction in intercellular space were observed. Fat accumulation was detected in the M and H groups, with the most pronounced steatosis in the M group. Blinded semiquantitative scoring of liver and colon H&E sections was performed by a hospital pathologist blinded to group allocation, using a 0–3 scale for hepatocyte injury, steatosis, inflammatory infiltration, mucosal damage, and goblet-cell loss. Two sections per animal and two fields per section were analyzed. The scores confirmed that the medium-dose group exhibited the most severe liver and colon injury, consistent with the biochemical and molecular results, and the between-group differences were statistically significant.

### 3.4. Expression of Tight Junction Proteins in the Colon

The colon H&E results suggested that combined Flu/Phe exposure induced colonic tissue injury. To further characterize the impact on intestinal barrier integrity, we quantified the mRNA and protein levels of the core tight junction proteins ZO-1, Occludin, and Claudin-1 by RT-qPCR and Western blot. Following combined Flu/Phe exposure, the transcriptional and protein abundances of all three proteins were markedly downregulated in rat colon tissue (***p*** < 0.05; Figure 5). Notably, ZO-1 and Claudin-1 exhibited the lowest expression in the M group, whereas Occludin reached its minimum in the H group. This divergence may reflect differential susceptibility of tight junction proteins to combined Flu/Phe exposure: ZO-1 and Claudin-1 may be more vulnerable to moderate toxic stress, whereas Occludin may require a higher toxicant burden to elicit comparable suppression. Alternative explanations—including biological variability, hormesis-like non-monotonic responses, or direct toxic suppression at distinct exposure concentrations—cannot be excluded and remain to be resolved by further mechanistic studies. Collectively, these data demonstrate that combined Flu/Phe exposure disrupts intestinal tight junction protein expression and consequently impairs gut barrier function [29].

### 3.5. Gut Microecological Diversity

We employed 16S rRNA gene sequencing to assess GM changes in the colon. Alpha diversity indices were used to assess species richness and evenness within each sample. Community richness was evaluated using ACE and Chao1 indices, and community diversity was assessed using Simpson and Shannon indices [30]. Compared with the C group, ACE, Chao1, Simpson, and Shannon indices were all significantly reduced in the exposure groups (***p*** < 0.05; Figure 6A–D), indicating that combined Flu/Phe exposure decreased GM diversity and richness.

Beta diversity was analyzed by NMDS, which reflects inter-sample differences as distances among points in multidimensional space [31]. NMDS analysis revealed significant separation (***p*** < 0.05) between the C group and the exposure groups (Figure 6E), demonstrating that combined Flu/Phe exposure alters the overall structure and composition of rat GM.

The GM composition at the phylum level is shown in Figure 6F. *Firmicutes*, *Bacteroidetes*, *Proteobacteria*, *Acidobacteriota*, *Actinobacteriota*, *Desulfobacteriota*, *Gemmatomonadota*, *Chloroflexi*, and *Campylobacteriota* were the most prevalent phyla. *Desulfobacterota*, *Gemmatimonadota*, *Chloroflexi*, and *Campylobacterota* were the dominant phyla shared across the four groups, collectively accounting for approximately 95% of the total abundance. Compared with the C group, the exposure groups exhibited a progressive increase in the relative abundance of seven Gram-negative phyla: *Bacteroidetes*, *Proteobacteria*, *Actinobacteriota*, *Desulfobacterota*, *Gemmatimonadota*, *Chloroflexi*, and *Campylobacterota.*

LEfSe analysis (LDA score > 3.0; detailed results summarized in Table S1) with an evolutionary branching diagram (Figure 6G,H) identified distinctive bacterial markers in each group. Enriched bacterial taxa in the C group included members of the order Clostridiales and the family Desulfovibrionaceae (genus Desulfovibrio). The L group was characterized by enrichment of the genus Lactobacillus (family Lactobacillaceae) and the genus Turicibacter. The M group was characterized by enrichment of members of the families Ruminococcaceae, Lachnospiraceae, and S24-7, and the genera Bacteroides, Prevotella, Phascolarctobacterium, and Sutterella. The H group was characterized by enrichment of the genus Lactobacillus and members of the order Actinomycetales (families Micrococcaceae and Corynebacteriaceae). These findings are consistent with the phylum-level composition results. Furthermore, under medium-dose exposure, Bacteroidetes (phylum) remained a dominant phylum and its subordinate order Bacteroidales was specifically enriched, suggesting that Bacteroidetes may play a key role in the toxicological response to medium-dose Flu and Phe exposure.

### 3.6. Effects on Gut Health

Given that Flu and Phe exposure significantly reduced GM diversity and remodeled community structure, we next examined the impact on gut health by analyzing abundance changes at the phylum level (Figure 7). The relative abundance of *Firmicutes* decreased significantly in a dose-dependent manner (***p*** < 0.05), reaching the lowest level in the H group. In contrast, the relative abundance of *Bacteroidetes* was significantly higher in the M group than in other groups (***p*** < 0.05), while no significant changes were observed in the L and H groups. Notably, the most pronounced microbial imbalance occurred in the M group and was strongly correlated with the degree of hepatic steatosis and inflammatory cell infiltration in this group. We hypothesize that the non-monotonic response of *Bacteroidetes*—peaking at the M dose and declining at the H dose—may reflect differential susceptibility of bacterial phyla to Flu and Phe. One possible explanation, consistent with previous reports [32] is that *Firmicutes* are more sensitive to moderate toxic stress, allowing *Bacteroidetes* to expand in relative abundance. Alternative explanations, including biological variability, hormesis-like responses, or direct toxic suppression at higher exposure levels, require further investigation through metagenomic or functional analyses.

### 3.7. LPS Translocation and Upregulation of the Hepatic TLR4 Signaling Pathway

Based on the observed gut barrier disruption and GM dysbiosis, we hypothesized that LPS serves as a critical mediator of gut–liver axis communication. To test this, we measured LPS concentrations in colon contents, serum, and liver. LPS levels were significantly elevated at all three sites in the exposure groups compared with the C group (***p*** < 0.05), with the highest levels observed in the M group (Figure 8A–C). This pattern of LPS elevation was consistent with the GM findings, particularly the peak relative abundance of Bacteroidetes in the M group (Figure 7B). Because 16S relative-abundance data do not quantify bacterial biomass or LPS production, and LPS bioactivity varies among taxa, these findings indicate an association between Gram-negative bacterial enrichment and elevated LPS rather than demonstrating a direct causal contribution.

Given that LPS exerts its pro-inflammatory effects primarily through TLR4, we examined hepatic TLR4 expression and its downstream signaling molecules, MyD88 and NLRP3. RT-qPCR and Western blot analyses (Figure 9) showed that the mRNA and protein expression of TLR4, MyD88, and NLRP3 were significantly upregulated in the exposure groups (***p*** < 0.05), with the highest levels in the M group, consistent with the LPS accumulation pattern (Figure 8C). Although these findings show increased mRNA and protein expression of TLR4, MyD88, and NLRP3, they do not directly demonstrate functional NLRP3 inflammasome activation, which would require additional evidence such as caspase-1 cleavage/activity, ASC oligomerization, and maturation of IL-1β; these functional endpoints remain to be assessed in future studies.

### 3.8. Liver Injury Induced by Flu and Phe Exposure

To assess the functional consequences of these molecular changes, we measured liver injury markers. Serum ALT and AST levels were significantly elevated in the M and H groups compared with the C group (***p*** < 0.05; Figure 10A,B), confirming hepatocellular injury following upregulation of the LPS–TLR4 signaling pathway. Notably, ALT and AST levels were significantly higher in the M group than in the H group (***p*** < 0.05). We hypothesize that this non-monotonic response may reflect dose-dependent cellular effects: at the highest exposure dose, severe hepatocellular injury may impair enzyme synthesis or release, resulting in lower serum transaminase levels despite substantial hepatic damage [33]. However, the precise mechanisms remain to be elucidated. Hepatic NEFA, T-CHO, and TG levels were also significantly elevated in the exposure groups compared with the C group (***p*** < 0.05; Figure 10C–E), with the most substantial increases in the M group, consistent with the most severe GM dysbiosis and the highest LPS levels observed in this group. Collectively, these data indicate that combined Flu/Phe exposure induces significant lipid accumulation in hepatocytes through disruption of the gut–liver axis, a pathological hallmark of non-alcoholic fatty liver disease (NAFLD).

### 3.9. Effects of Flu and Phe Exposure on Hepatic Inflammation and Oxidative Stress

Inflammation and oxidative stress are recognized as key contributors to the pathophysiology of NAFLD. Following combined Flu/Phe exposure (Figure 11A–I), the hepatic levels of the pro-inflammatory cytokines IL-6, IL-1β, and TNF-α showed an increasing trend, with the most pronounced elevation in the M group, indicating that the hepatic inflammatory response was strongest in this group. Meanwhile, the anti-inflammatory cytokine IL-10 was also significantly increased in the exposure groups (***p*** < 0.05), with higher levels in the L and H groups compared with the M group. We hypothesize that the lower IL-10 level in the M group, despite the most intense pro-inflammatory response, may reflect a dose-dependent shift in the balance between pro- and anti-inflammatory pathways. One possible explanation is that moderate-dose exposure preferentially activates pro-inflammatory signaling, whereas at low or high doses, additional regulatory mechanisms may be engaged to modulate the inflammatory response. Further studies, including analysis of regulatory T cell markers and dynamic cytokine profiling, are required to test these hypotheses.

Regarding oxidative stress, SOD activity, a key indicator of antioxidant capacity, was significantly decreased in the exposure groups (***p*** < 0.05; Figure 11J). Conversely, MDA (a lipid peroxidation product) and 8-OHdG (a marker of oxidative DNA damage) were significantly elevated (***p*** < 0.05; Figure 11K,L). These changes collectively indicate a marked reduction in hepatic antioxidant capacity following combined Flu/Phe exposure.

### 3.10. Effects of Combined Flu/Phe Exposure on Hepatic Lipid Metabolism

To systematically investigate hepatic lipid metabolism, we performed Oil Red O staining, RT-qPCR, and Western blot analyses. Oil Red O staining (Figure 12A) revealed abundant red lipid droplets in the livers of the exposure groups. ImageJ (Fiji Is Just ImageJ, version 2.16.0/1.54p, Java 21.0.7; National Institutes of Health, Bethesda, MD, USA) quantification (Figure 12B) confirmed that the lipid droplet area was significantly increased in the exposure groups compared with the C group (***p*** < 0.05), with the most severe accumulation in the M group. We next examined key regulators of hepatic lipid metabolism: PPAR-α (promotes fatty acid catabolism), PPAR-γ (regulates lipid storage, differentiation, and insulin sensitivity), and FABP1 (involved in lipid transport and metabolism). Compared with the C group (Figure 12C–I), PPAR-α expression was decreased in the L and H groups but increased in the M group (***p*** < 0.05). PPAR-γ expression showed an increasing trend across the exposure groups, with the most significant increase in the M group (***p*** < 0.05). FABP1 expression was increased in the M and H groups (***p*** < 0.05), with the most significant increase in the M group. Together with the Oil Red O staining, these data indicate that the M group exhibited the most pronounced lipid metabolism dysregulation and lipid accumulation.

### 3.11. Effect of Combined Flu/Phe Exposure on Gut–Liver Axis Metabolomics

Given that the M group exhibited the most pronounced gut–liver axis injury pattern, we focused on the C and M groups for metabolomics analysis. PLS-DA revealed clear separation between the C and M groups for both colon content metabolites (R^2^Y = 0.999, Q^2^ = 0.921) and liver metabolites (R^2^Y = 0.996, Q^2^ = 0.967) (Figure 13A,B), indicating that combined Flu/Phe exposure induced significant metabolic alterations in both the gut and liver. To assess overfitting, permutation testing (200 permutations) was performed; the permutation intercepts were R^2^ = 0.85 and Q^2^ = −0.25 for colon, and R^2^ = 0.82 and Q^2^ = −0.28 for liver. The negative Q^2^ intercepts indicate that the models were not overfitted. Cross-validation was performed using 7-fold cross-validation. We nevertheless acknowledge that the small sample size (*n* = 5 per group) limits the generalizability of the supervised models, and the metabolite differences should therefore be interpreted as exploratory. PCA further confirmed clear separation between colon contents and liver samples in the C and M groups (Figure 13C,D). Differential metabolite analysis identified 403 significantly altered metabolites in colon contents (101 decreased, 302 increased; Figure 13E) and 939 in the liver (406 decreased, 533 increased; Figure 13F). KEGG pathway enrichment analysis identified 20 key metabolic pathways in the colon and liver (Figure 13G,H). Notably, bile secretion was the most significantly enriched pathway shared by the colon and liver. The differential metabolite–pathway network (Figure 13I) revealed that the bile secretion pathway is linked to a substantial number of differential metabolites and interacts extensively with multiple other metabolic pathways. Collectively, these findings identify bile secretion as a major hub for metabolic abnormalities in the gut–liver axis during Flu and Phe exposure.

### 3.12. Effects of Combined Flu/Phe Exposure on Bile Acid Metabolism in the Gut–Liver Axis

KEGG pathway analysis identified bile secretion as a key nexus for metabolic disruption in the gut–liver axis following combined Flu/Phe exposure. Since bile acids are the primary functional constituents of bile [29], we further analyzed alterations in bile acid profiles. By metabolomics, we identified 31 bile acid metabolites. These were putatively annotated (Metabolomics Standards Initiative level 2) based on accurate mass and database matching, without authentic-standard or MS/MS confirmation; their individual identities and quantities therefore require targeted validation. Volcano plot analysis revealed that 9 metabolites were upregulated and 4 were downregulated in colon contents (Figure 14A). Among the upregulated metabolites, two species annotated as deoxycholic acid glycine conjugate and taurodeoxycholic acid showed a positive correlation with the relative abundance of Gram-negative bacteria (*Bacteroidetes*) in the colon. Two metabolites annotated as cholyltyrosine and cholylglutamine, conjugated bile-acid metabolites of bile acid metabolism, were also detected. In the M group, four metabolites—annotated as 12β-hydroxy-5β-cholanoic acid, 3α,7α-dihydroxy-5β-cholestanate, sulfoglycolithocholate(2-), and 12α-hydroxy-3-oxocholadienic acid—were decreased relative to the C group (Figure 14A). Correlation heat map analysis (Figure 14C) demonstrated that the metabolites annotated as deoxycholic acid glycine conjugate, taurodeoxycholic acid, cholyltyrosine, cholylglutamine, and 7β-hydroxy-3-oxo-5β-cholanoic acid were significantly positively correlated with each other (***p*** < 0.05), and significantly negatively correlated (***p*** < 0.05) with the four decreased metabolites.

In the liver, volcano plot analysis identified 8 upregulated and 9 downregulated metabolites (Figure 14B). The upregulated metabolites included annotated as chenodeoxycholic acid, 12α-hydroxy-3-oxocholadienic acid, nordoxycholic acid, 12β-hydroxy-5β-cholanoic acid, 3α,7α-dihydroxy-5β-cholestanate, chenodeoxycholylvaline, sulfoglycolithocholate(2-), and 24-hydroxycholesterol. The downregulated metabolites included cholyltyrosine, 22α-hydroxy-campest-4-en-3-one, 3β,7α-dihydroxy-5-cholestenoate, cholylglutamate, 7β-hydroxy-3-oxo-5β-cholanoic acid, and taurolithocholic acid. Correlation heat map analysis (Figure 14D) showed that the upregulated metabolites were significantly positively correlated with each other (***p*** < 0.05), and significantly negatively correlated with the downregulated metabolites.

To further characterize the signaling pathways underlying these bile acid changes, we examined the hepatic FXR/SHP signaling axis. Following combined Flu/Phe exposure, the mRNA and protein expression of FXR and SHP was significantly decreased (***p*** < 0.05; Figure 14E–M). In contrast, the mRNA and protein expression of CYP7A1 and CYP8B1 was significantly increased (***p*** < 0.05; Figure 14E–M). These results demonstrate that combined Flu/Phe exposure suppresses FXR/SHP signaling and upregulates CYP7A1 and CYP8B1 expression in the liver.

## 4. Discussion

In this study, we established a rat model of combined Flu/Phe exposure validated by urinary 2-OH Flu and 9-OH Phe concentrations and colonic and hepatic AHR/CYP1A1 expression. Previous studies have demonstrated a strong association between urinary 2-OH Flu/9-OH Phe and the magnitude of Flu/Phe exposure [34,35], and AHR/CYP1A1 expression reflects both exposure status and the toxicological effects of these compounds [36,37]. The dose-dependent elevation of these markers therefore confirms successful exposure modeling and an enhancement of stress responses, including inflammation and oxidative stress [38].

The intestine is the primary absorptive organ, and our data suggest that combined Flu/Phe exposure promotes liver injury through disruption of the gut barrier and gut-microbiota balance via the gut–liver axis. The reduced expression of ZO-1, Occludin, and Claudin-1 is consistent with gut-barrier impairment reported previously [39]. We hypothesize that this damage may be mediated by colonic AHR activation, which drives CYP1A1-mediated conversion of Flu/Phe into intermediate metabolites that may compromise tight-junction proteins [40]; this hypothesis, however, requires direct experimental validation. The accompanying gut-microbiota dysbiosis, characterized by an increased proportion of Gram-negative bacteria and reduced beneficial bacteria, likely further exacerbates gut-barrier damage.

Consistent with the expansion of Gram-negative taxa, LPS was elevated in colon contents, serum, and liver of the exposure groups [41]. We observed upregulated hepatic expression of TLR4, MyD88, and NLRP3, which was associated with elevated IL-6, IL-1β, and TNF-α [42]; notably, we did not measure caspase-1 cleavage, ASC oligomerization, or IL-1β maturation, so functional NLRP3 inflammasome activation was not directly demonstrated. The reduced IL-10 in the M group, which showed the strongest pro-inflammatory response, may reflect a pro-/anti-inflammatory imbalance, possibly through NF-κB hyperactivation [43]. The accompanying oxidative stress (reduced SOD, elevated MDA and 8-OHdG) is consistent with IL-1β/TNF-α-driven NADPH oxidase activation [44], although direct mitochondrial toxicity of Flu/Phe metabolites remains an alternative explanation that requires further study.

Inflammatory responses and oxidative stress frequently lead to hepatic lipid metabolism disorders [45,46]. The increased PPAR-γ, which promotes lipid storage, is consistent with the observed lipid accumulation, whereas the altered PPAR-α and FABP1 responses may reflect a compensatory reaction to excessive lipid accumulation in the M group; the decreased PPAR-α in the H group may reflect mitochondrial dysfunction or impaired fatty-acid oxidation [39].

Beyond inducing liver injury through gut-microbiota alteration, PAHs may also exacerbate liver injury by disrupting metabolic homeostasis [16,25,35]. Our metabolomic analysis of the control and medium-dose groups identified bile-acid metabolism as the most prominently affected pathway in the gut–liver axis, and the suppressed FXR/SHP expression together with upregulated CYP7A1/CYP8B1 is consistent with disrupted bile-acid feedback regulation. However, because the bile-acid species were identified by untargeted metabolomics without authentic-standard confirmation, and metabolomic profiling was restricted to the control and medium-dose groups, the causal contribution of specific bile-acid species to liver injury remains to be established.

We distinguish between the mechanisms directly demonstrated by our experiments and those that remain hypothetical. Our experiments directly demonstrate that combined Flu/Phe exposure disrupted the gut barrier, induced gut-microbiota dysbiosis, elevated LPS, upregulated hepatic TLR4/MyD88/NLRP3 expression, altered the FXR/SHP/CYP7A1/CYP8B1 axis, and induced hepatic inflammation, oxidative stress, and lipid accumulation. By contrast, several mechanistic links are inferred from associations rather than directly proven: (i) the causal contribution of Gram-negative bacteria to LPS elevation; (ii) functional NLRP3 inflammasome activation; and (iii) the causal contribution of bile-acid dysregulation to liver injury. Confirming these inferred links will require targeted interventions, such as LPS neutralization, FXR/SHP pathway modulation, or germ-free models.

Several limitations of this study should be acknowledged. We employed a broad-spectrum untargeted metabolomics approach; consequently, the precise identities and quantities of specific bile acids remain to be fully characterized, and targeted metabolomics with authentic standards will be essential in future studies. Metabolomic profiling was restricted to the control and medium-dose groups, which prevents evaluation of whether bile-acid changes follow the proposed non-monotonic dose response. We also observed that the medium-dose group exhibited stronger effects than the high-dose group across several endpoints; although this is suggestive of a non-monotonic dose response, it does not by itself establish such a relationship, and alternative explanations—including sampling variation and biological variability—cannot be excluded. Finally, only male Wistar rats were used to avoid the confounding protective effects of estrogens on toxicant-induced fatty liver, which limits the generalizability of our findings to females. Further studies—including germ-free animal models, LPS neutralization experiments, and FXR/SHP pathway-specific interventions—are required to distinguish among these possibilities. Although we characterized the consequences of gut-microbiota dysbiosis, we did not directly measure the microbial biotransformation of fluorene and phenanthrene. Metagenomic or functional analyses of microbial PAH-metabolizing activity will be required in future studies to determine the contribution of microbial biotransformation to the observed effects.

## 5. Conclusions

In summary, this study demonstrates that combined Flu/Phe exposure induces GM disruption—characterized by an increased proportion of Gram-negative bacteria—and impairs intestinal barrier integrity, enabling LPS to translocate to the liver through the gut–liver axis. Hepatic LPS then upregulates the TLR4/MyD88/NLRP3 signaling axis, which was associated with inflammation, oxidative stress, and lipid metabolism disorders. Concurrently, combined Flu/Phe exposure disrupts bile acid metabolism through suppression of the FXR/SHP axis and upregulation of CYP7A1 and CYP8B1, further exacerbating liver injury. The medium-dose group exhibited the strongest phenotype across multiple endpoints (M > H > L); however, whether this reflects a true non-monotonic dose response requires formal dose–response modeling and cannot be concluded from the observed group differences alone. This dose may be useful for subsequent mechanistic validation studies, such as fecal microbiota transplantation (FMT), LPS neutralization, or TLR4 inhibition. A graphical summary integrating these multi-omics findings into a comprehensive mechanistic model of LMW-PAH-induced liver injury via the gut–liver axis is presented in Figure 15. Collectively, these findings provide a mechanistic framework for LMW-PAH-induced liver injury and identify potential therapeutic targets—including Gram-negative bacteria, the TLR4 pathway, and the FXR/SHP axis—for the prevention and treatment of LMW-PAH-associated hepatic damage. The exposure doses here exceed realistic dietary intake, so this study should be interpreted as a high-dose mechanistic toxicology model rather than a model of environmentally or dietary relevant exposure. Whether the identified molecular nodes can serve as biomarkers for risk assessment of LMW-PAH contamination will require validation at dietary-relevant exposure levels.

## Figures and Tables

**Figure 1:**
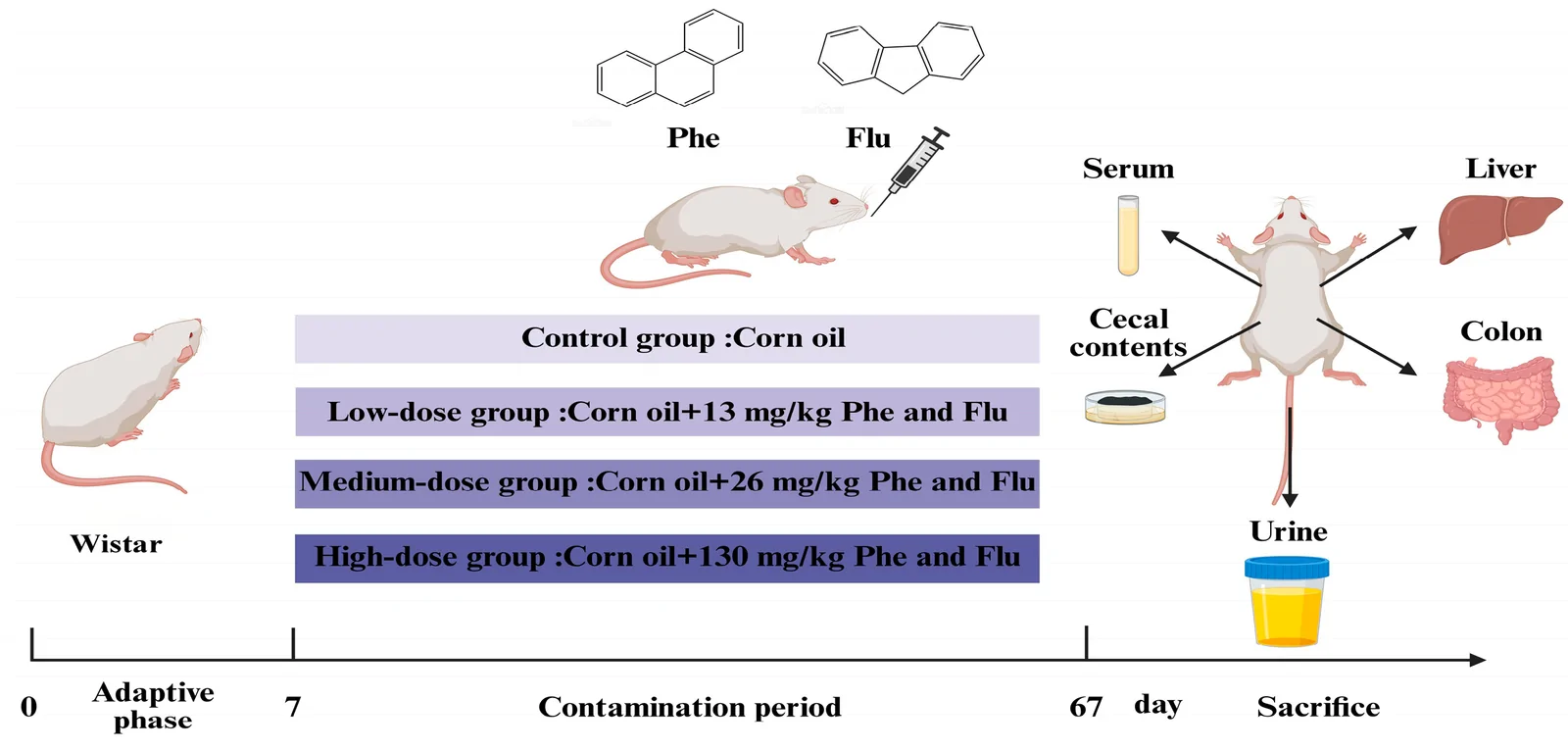
Schematic diagram of the experimental design. C, control group (corn oil); L, low-dose group (13 mg/kg/day each of Flu and Phe); M, medium-dose group (26 mg/kg/day each); H, high-dose group (130 mg/kg/day each).

**Figure 2:**
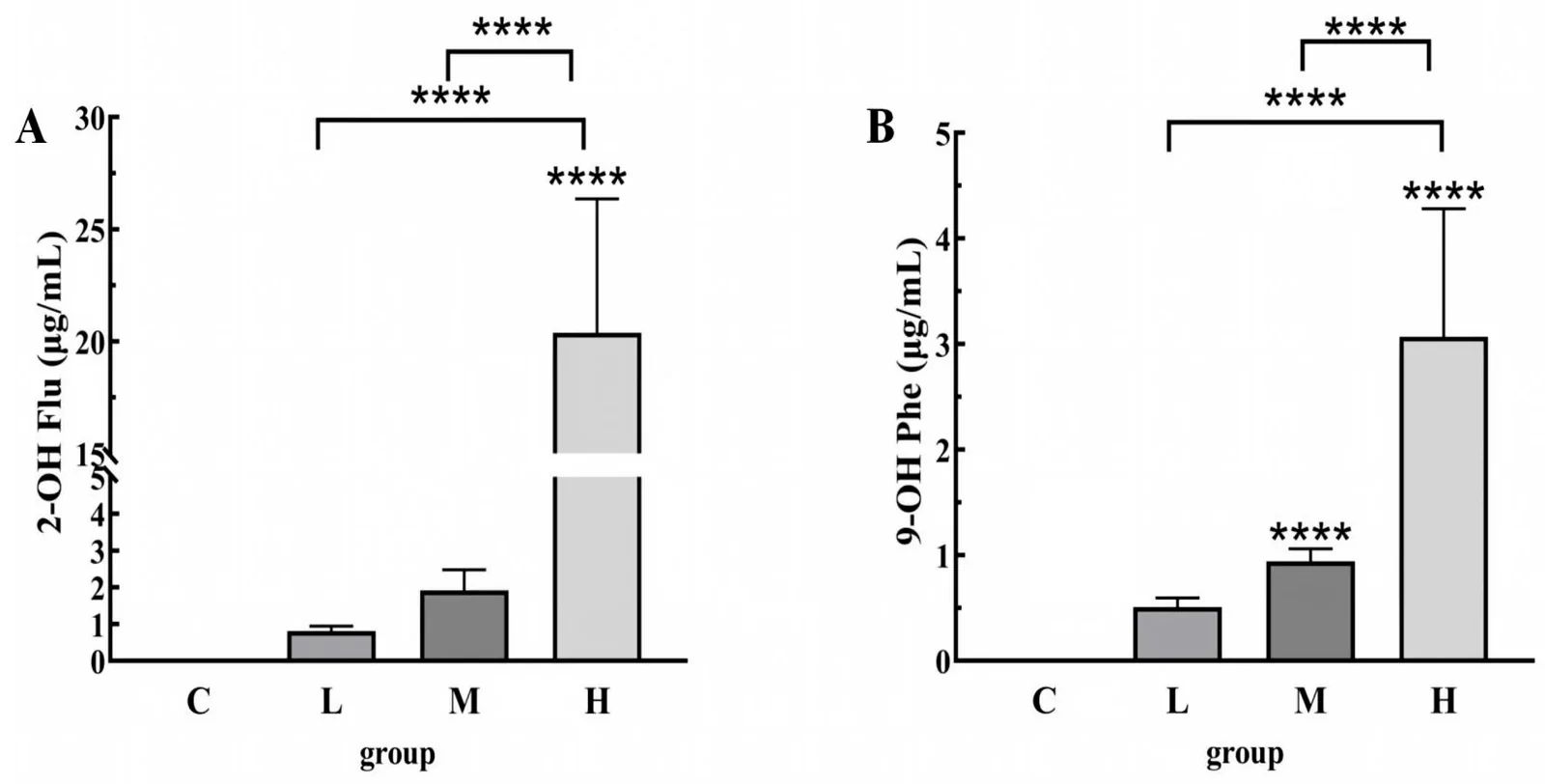
Effect of combined Flu/Phe exposure on urinary levels of 2-OH Flu (**A**) and 9-OH Phe (**B**). Data are presented as mean ± SD (*n* = 8 rats per group). Statistical significance was determined by one-way ANOVA followed by Tukey’s test. **** ***p*** < 0.0001 compared with the C group. All analyses were performed blinded to group allocation. C, control; L, low dose; M, medium dose; H, high dose.

**Figure 3:**
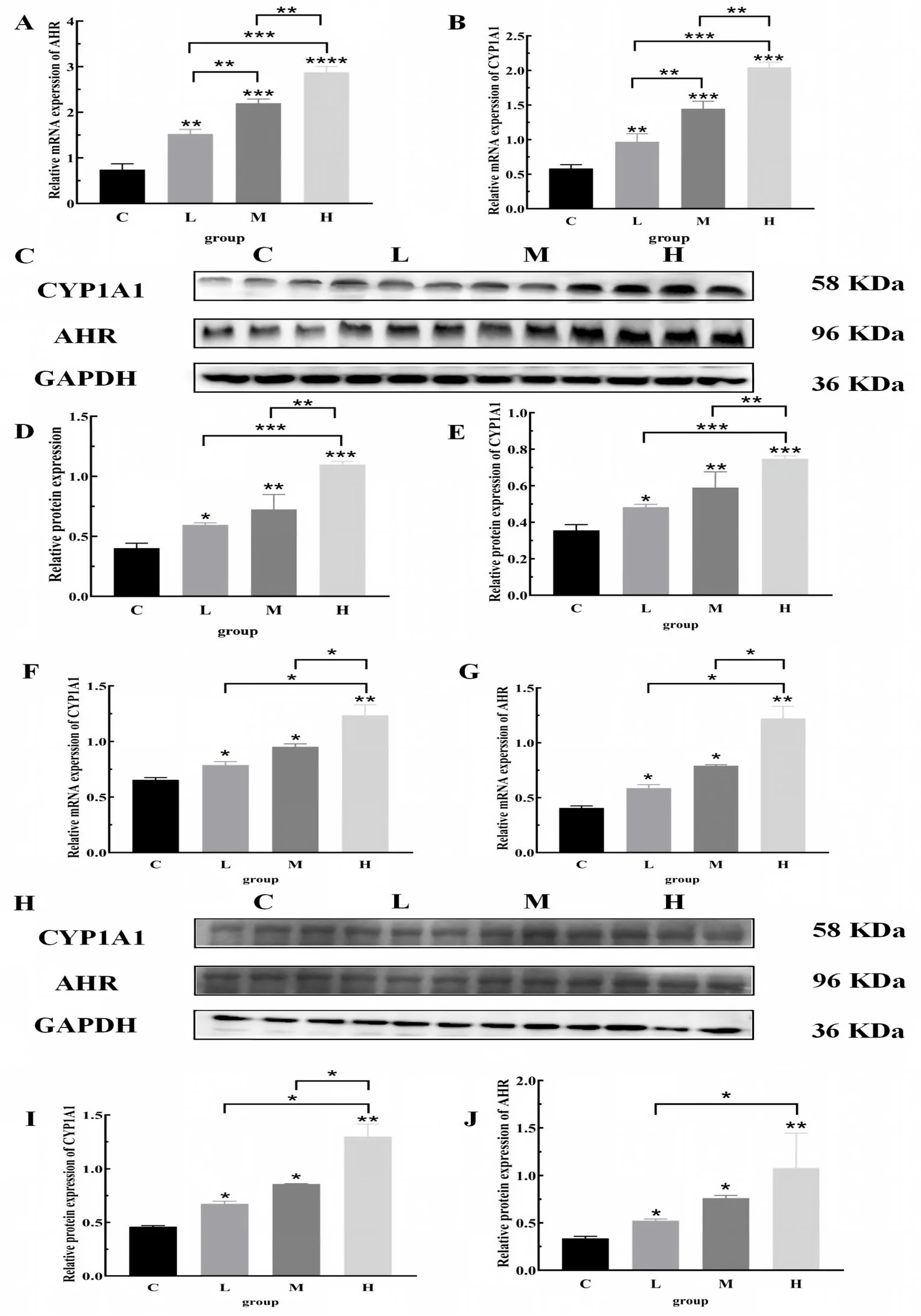
Effect of combined Flu/Phe exposure on mRNA and protein levels of AHR and CYP1A1 in the colon and liver. (**A**) Colon AHR mRNA. (**B**) Colon CYP1A1 mRNA. (**C**) Colon Western blot bands. (**D**) Colon AHR protein (semi-quantitative). (**E**) Colon CYP1A1 protein (semi-quantitative). (**F**) Liver AHR mRNA. (**G**) Liver CYP1A1 mRNA. (**H**) Liver Western blot bands. (**I**) Liver AHR protein (semi-quantitative). (**J**) Liver CYP1A1 protein (semi-quantitative). Data are presented as mean ± SD (*n* = 5 rats per group). Statistical significance was determined by one-way ANOVA followed by Tukey’s test. * ***p*** < 0.05, ** ***p*** < 0.01, *** ***p*** < 0.001, **** ***p*** < 0.0001 compared with the C group. All analyses were performed blinded to group allocation. C, control; L, low dose; M, medium dose; H, high dose.

**Figure 4:**
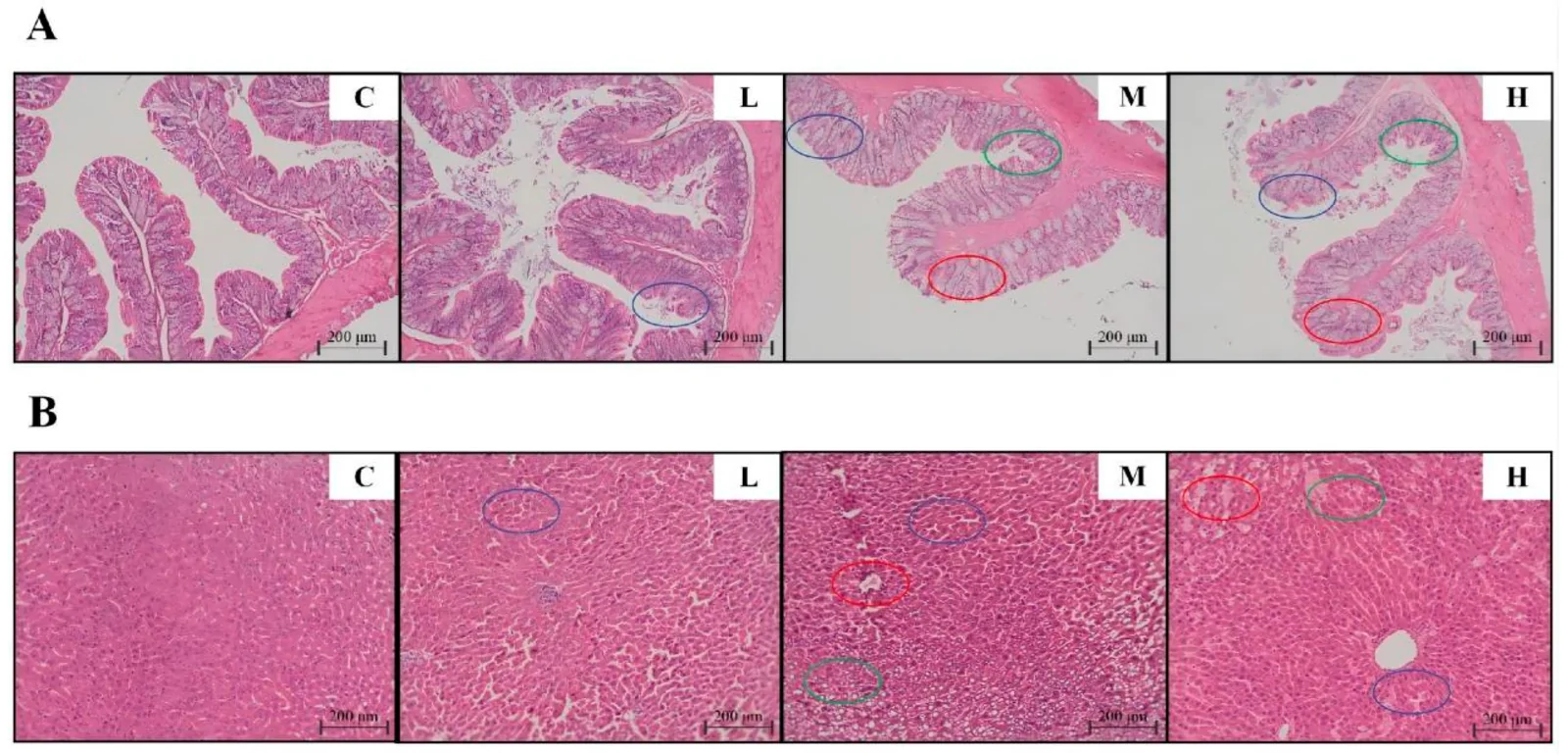
Histopathological alterations in colon and liver following combined Flu/Phe exposure. (**A**) Colon H&E (100×). Blue, red, and green markers indicate mucosal structural damage, abnormal cell morphology, goblet cell loss, and crypt distortion, respectively. (**B**) Liver H&E (100×). Blue, red, and green markers indicate narrowed intercellular space, inflammatory infiltration, and fat accumulation, respectively. *n* = 3 per group. Blinded semiquantitative scoring (0–3) was performed on two sections and two fields per animal by a pathologist blinded to group allocation. C, control; L, low dose; M, medium dose; H, high dose.

**Figure 5:**
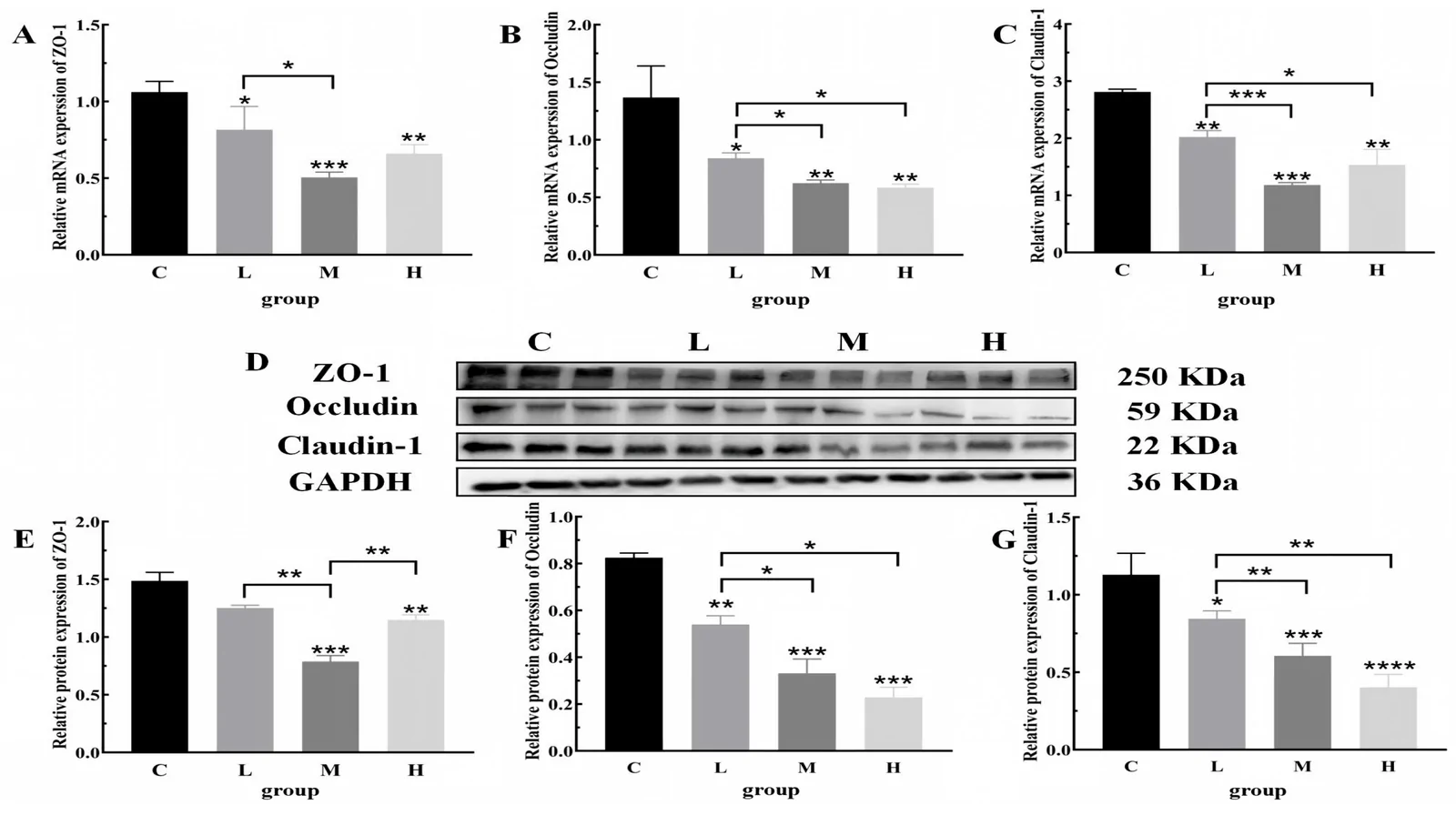
Effect of combined Flu/Phe exposure on colonic tight junction proteins. (**A**) ZO-1 mRNA. (**B**) Occludin mRNA. (**C**) Claudin-1 mRNA. (**D**) Western blot bands. (**E**) ZO-1 protein (semi-quantitative). (**F**) Occludin protein (semi-quantitative). (**G**) Claudin-1 protein (semi-quantitative). Data are presented as mean ± SD (*n* = 5 rats per group). Statistical significance was determined by one-way ANOVA followed by Tukey’s test. * ***p*** < 0.05, ** ***p*** < 0.01, *** ***p*** < 0.001, **** ***p*** < 0.0001compared with the C group. All analyses were performed blinded to group allocation. C, control; L, low dose; M, medium dose; H, high dose.

**Figure 6:**
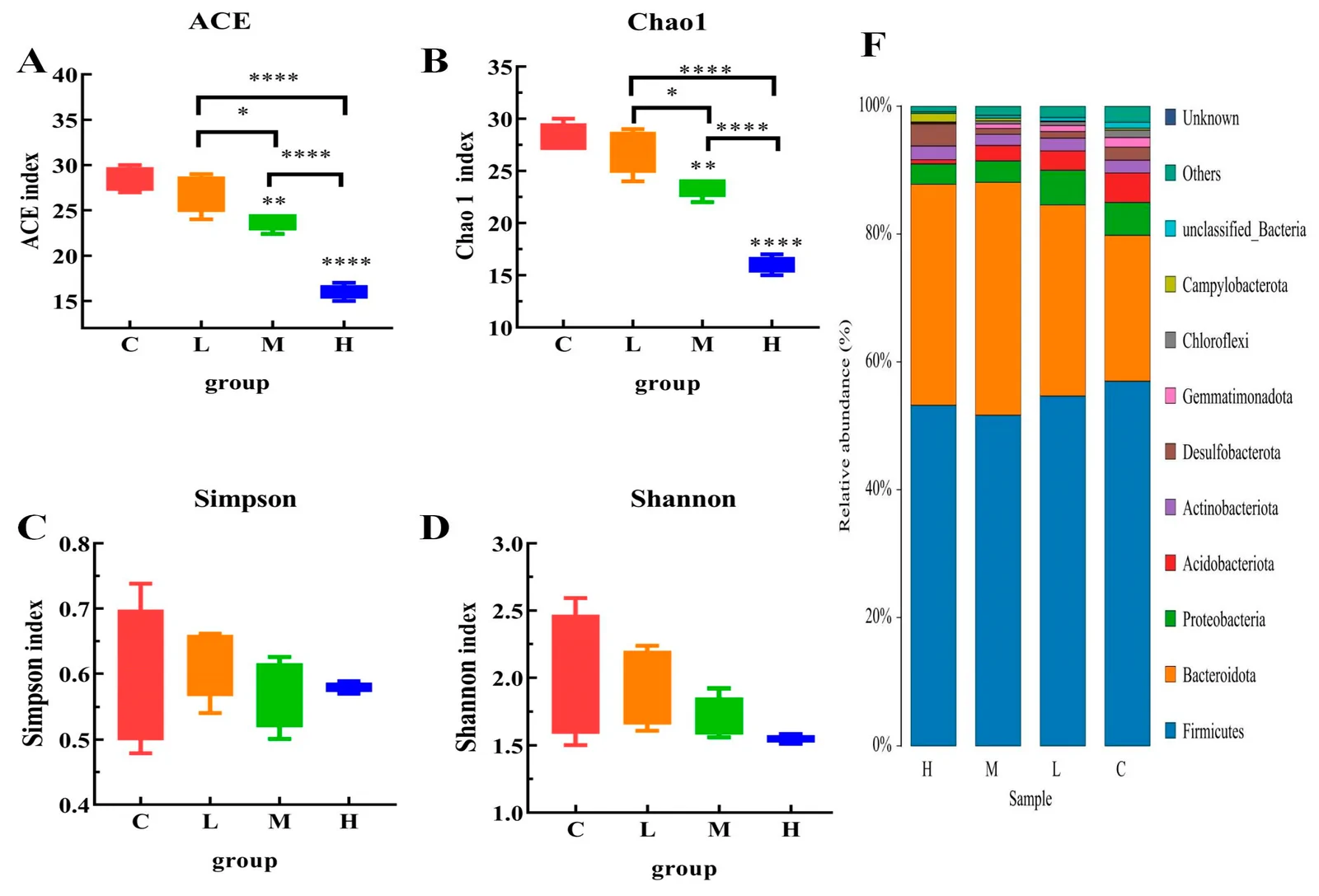

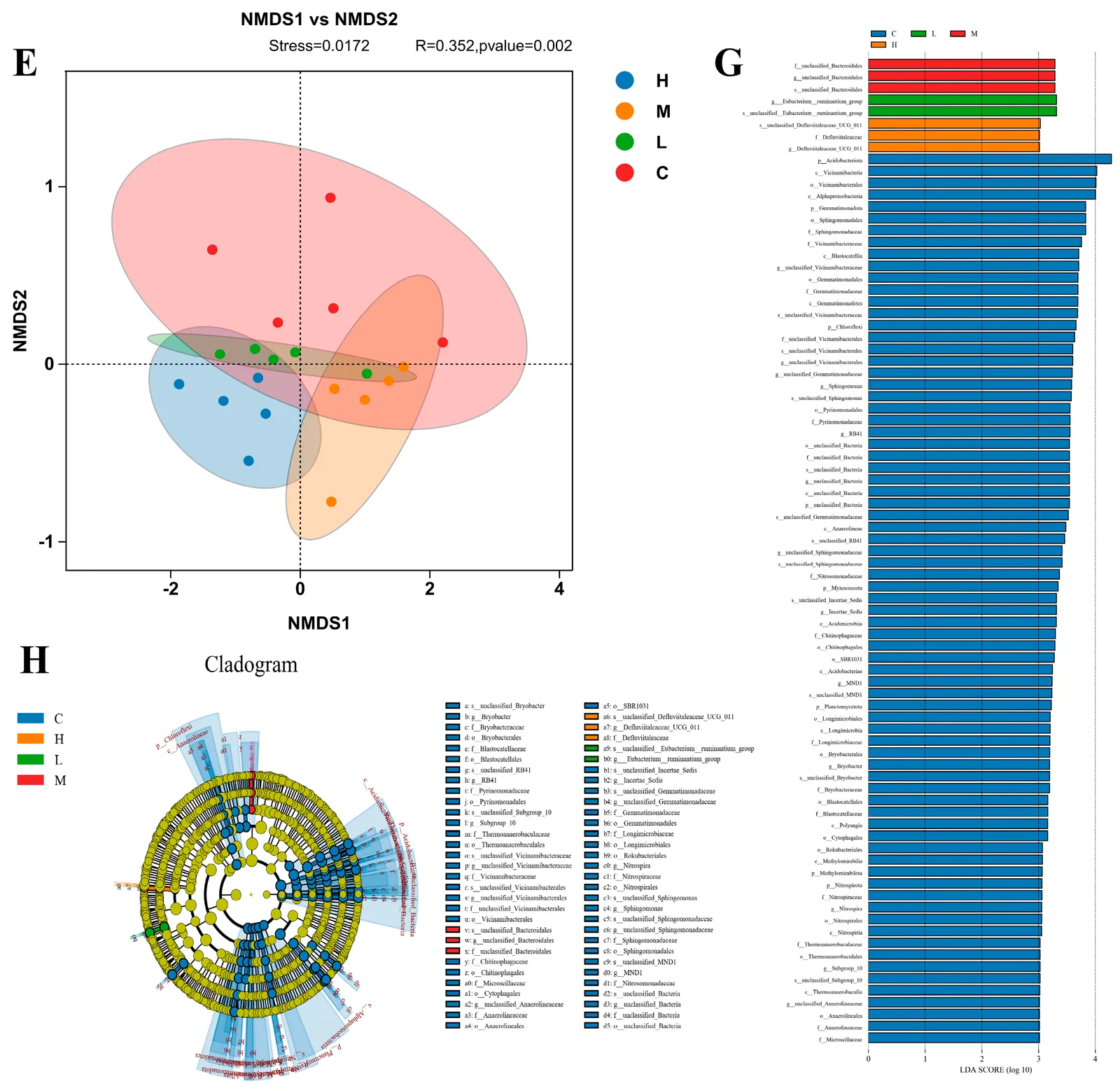
Effects of combined Flu/Phe exposure on GM diversity. (**A**–**D**) Alpha diversity indices. (**E**) Beta diversity (NMDS). (**F**) Phylum-level GM composition. (**G**) LDA effect size histogram (LDA > 3.0). (**H**) Cladogram illustrating taxonomic differences among groups. Data are presented as mean ± SD (*n* = 5 rats per group). Statistical significance for α-diversity was determined by one-way ANOVA followed by Tukey’s test; β-diversity was analyzed by PERMANOVA. * ***p*** < 0.05, ** ***p*** < 0.01, **** ***p*** < 0.0001 compared with the C group. All analyses were performed blinded to group allocation. C, control; L, low dose; M, medium dose; H, high dose.

**Figure 7:**
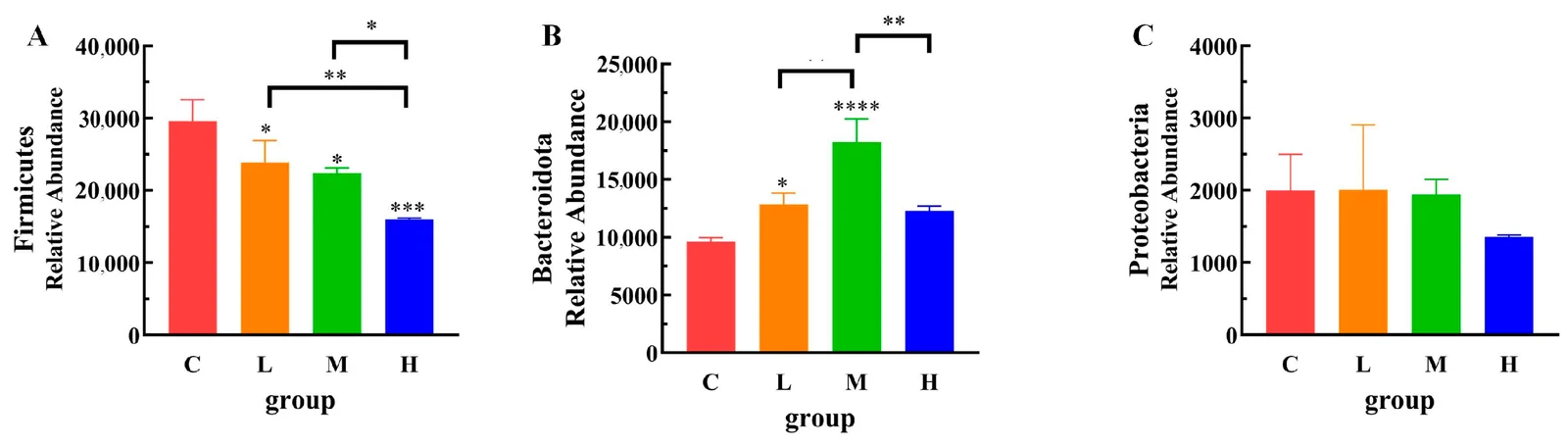
Effects of combined Flu/Phe exposure on gut health. Relative abundance of microorganisms at the phylum level was analyzed using the Mann–Whitney U test. (**A**) Firmicutes. (**B**) Bacteroidetes. (**C**) Proteobacteria. Data are presented as mean ± SD (*n* = 5 rats per group). * ***p*** < 0.05, ** ***p*** < 0.01, *** ***p*** < 0.001, **** ***p*** < 0.0001 compared with the C group. All analyses were performed blinded to group allocation. C, control; L, low dose; M, medium dose; H, high dose.

**Figure 8:**
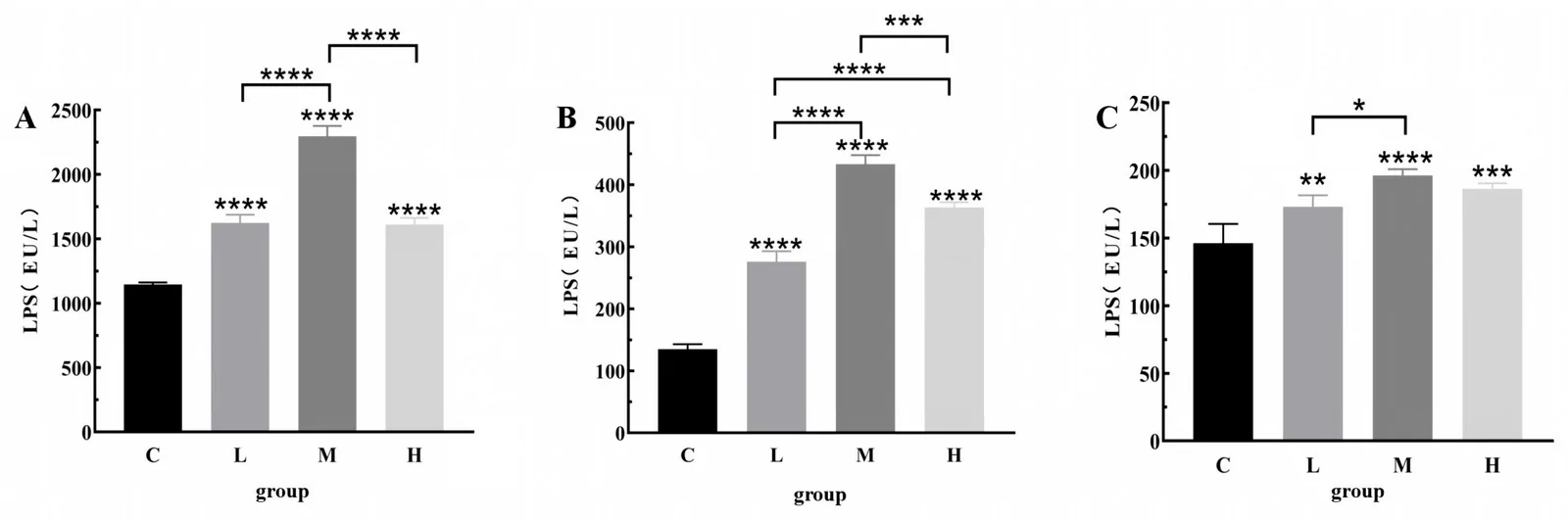
Effect of combined Flu/Phe exposure on LPS translocation. (**A**) LPS in colon contents. (**B**) LPS in serum. (**C**) LPS in liver. Data are presented as mean ± SD (*n* = 5 rats per group). Statistical significance was determined by one-way ANOVA followed by Tukey’s test. * ***p*** < 0.05, ** ***p*** < 0.01, *** ***p*** < 0.001, **** ***p*** < 0.0001 compared with the C group. All analyses were performed blinded to group allocation. C, control; L, low dose; M, medium dose; H, high dose.

**Figure 9:**
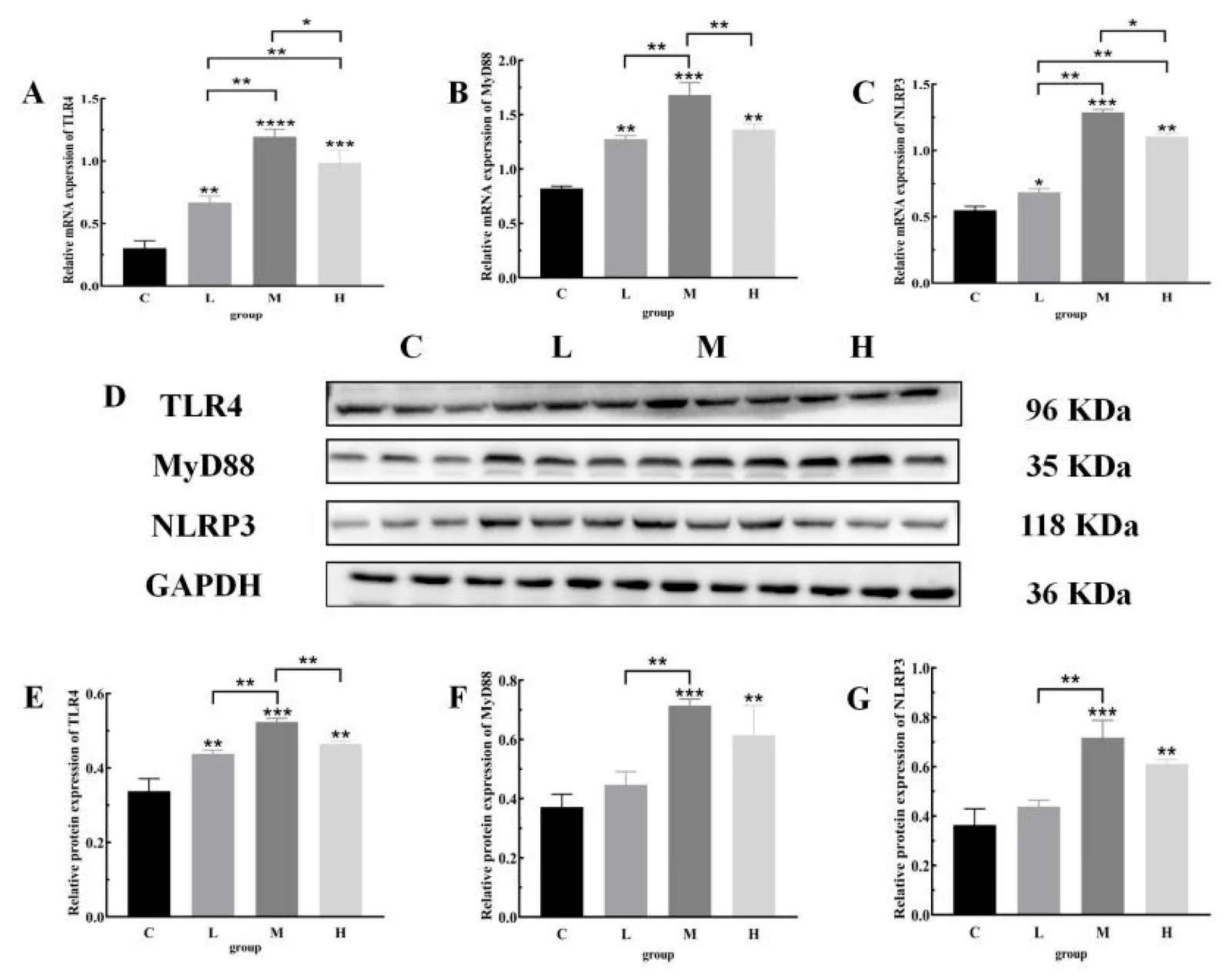
Effects of combined Flu/Phe exposure on the hepatic TLR4/MyD88/NLRP3 signaling pathway. (**A**) TLR4 mRNA. (**B**) MyD88 mRNA. (**C**) NLRP3 mRNA. (**D**) Western blot bands. (**E**) TLR4 protein (semi-quantitative). (**F**) MyD88 protein (semi-quantitative). (**G**) NLRP3 protein (semi-quantitative). Data are presented as mean ± SD (*n* = 5 rats per group). Statistical significance was determined by one-way ANOVA followed by Tukey’s test. * ***p*** < 0.05, ** ***p*** < 0.01, *** ***p*** < 0.001, **** ***p*** < 0.0001 compared with the C group. All analyses were performed blinded to group allocation. C, control; L, low dose; M, medium dose; H, high dose.

**Figure 10:**
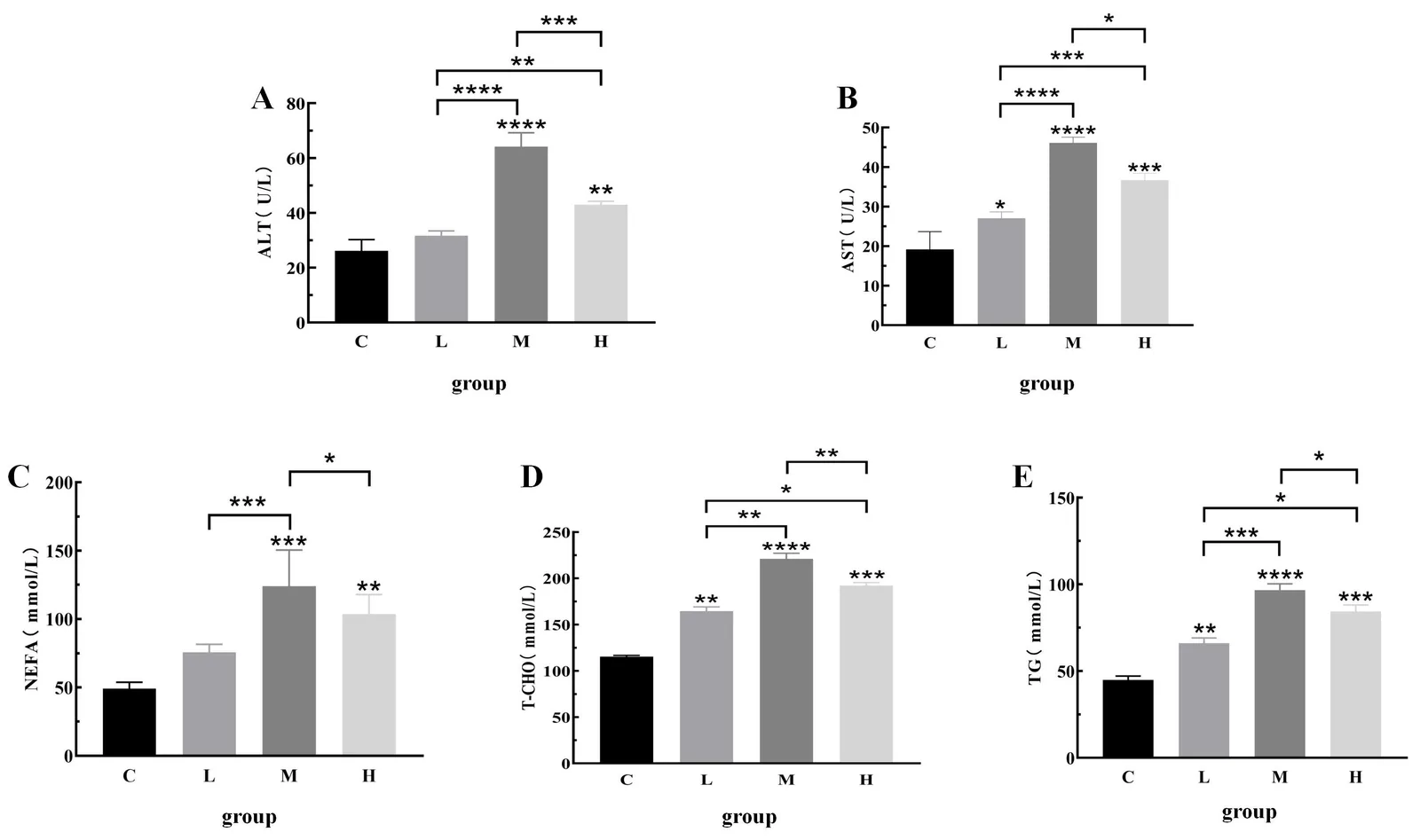
Effect of combined Flu/Phe exposure on liver injury markers. (**A**) Serum ALT. (**B**) Serum AST. (**C**) Liver NEFA. (**D**) Liver T-CHO. (**E**) Liver TG. Data are presented as mean ± SD (*n* = 5 rats per group). Statistical significance was determined by one-way ANOVA followed by Tukey’s test. * ***p*** < 0.05, ** ***p*** < 0.01, *** ***p*** < 0.001, **** ***p*** < 0.0001 compared with the C group. All analyses were performed blinded to group allocation. C, control; L, low dose; M, medium dose; H, high dose.

**Figure 11:**
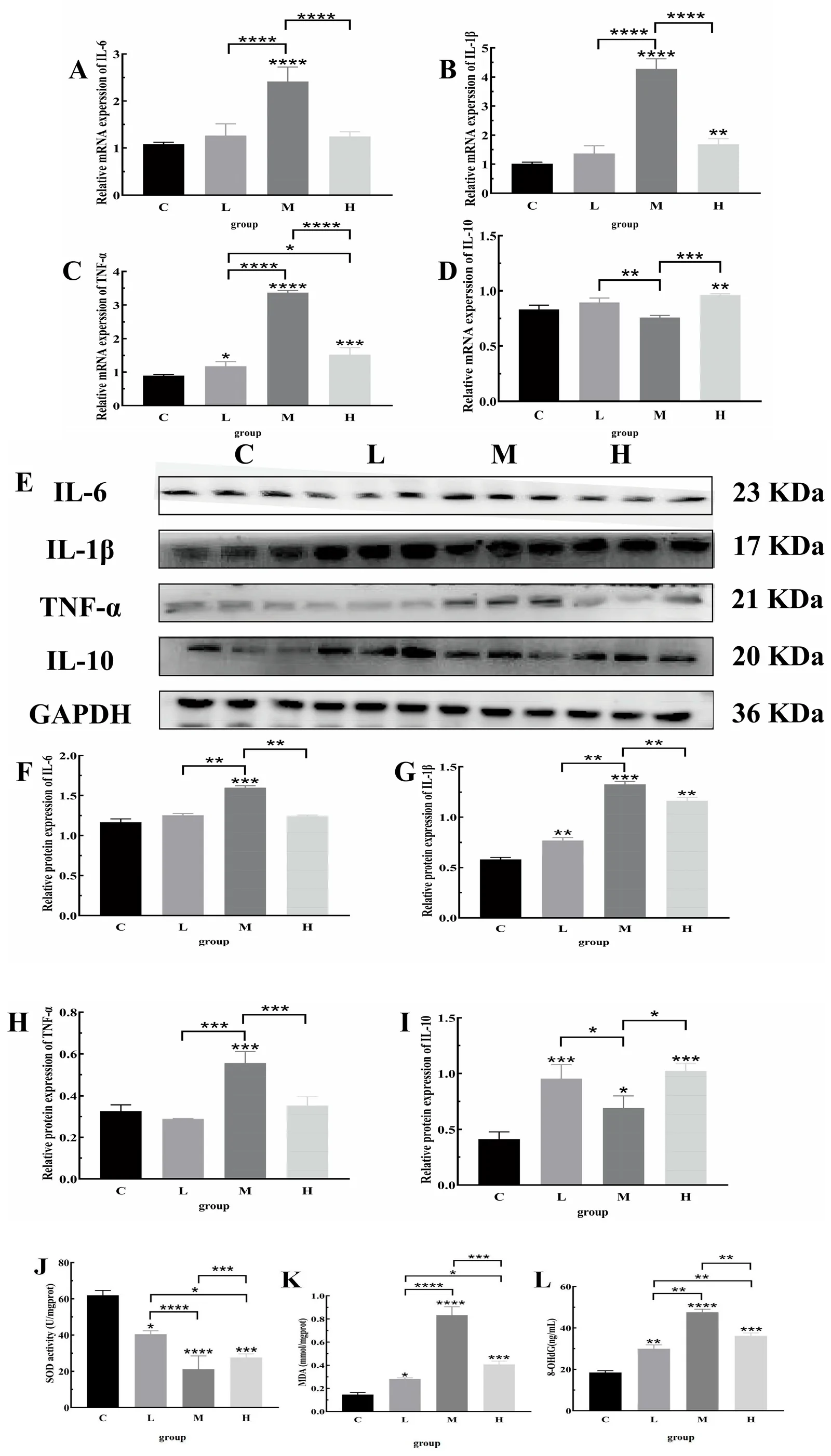
Effect of combined Flu/Phe exposure on hepatic inflammation and oxidative stress. (**A**) IL-6 mRNA. (**B**) IL-1β mRNA. (**C**) TNF-α mRNA. (**D**) IL-10 mRNA. (**E**) Western blot bands. (**F**) IL-6 protein (semi-quantitative). (**G**) IL-1β protein (semi-quantitative). (**H**) TNF-α protein (semi-quantitative). (**I**) IL-10 protein (semi-quantitative). (**J**) Liver SOD. (**K**) Serum MDA. (**L**) Liver 8-OHdG. Data are presented as mean ± SD (*n* = 5 rats per group). Statistical significance was determined by one-way ANOVA followed by Tukey’s test. * ***p*** < 0.05, ** ***p*** < 0.01, *** ***p*** < 0.001, **** ***p*** < 0.0001 compared with the C group. All analyses were performed blinded to group allocation. C, control; L, low dose; M, medium dose; H, high dose.

**Figure 12:**
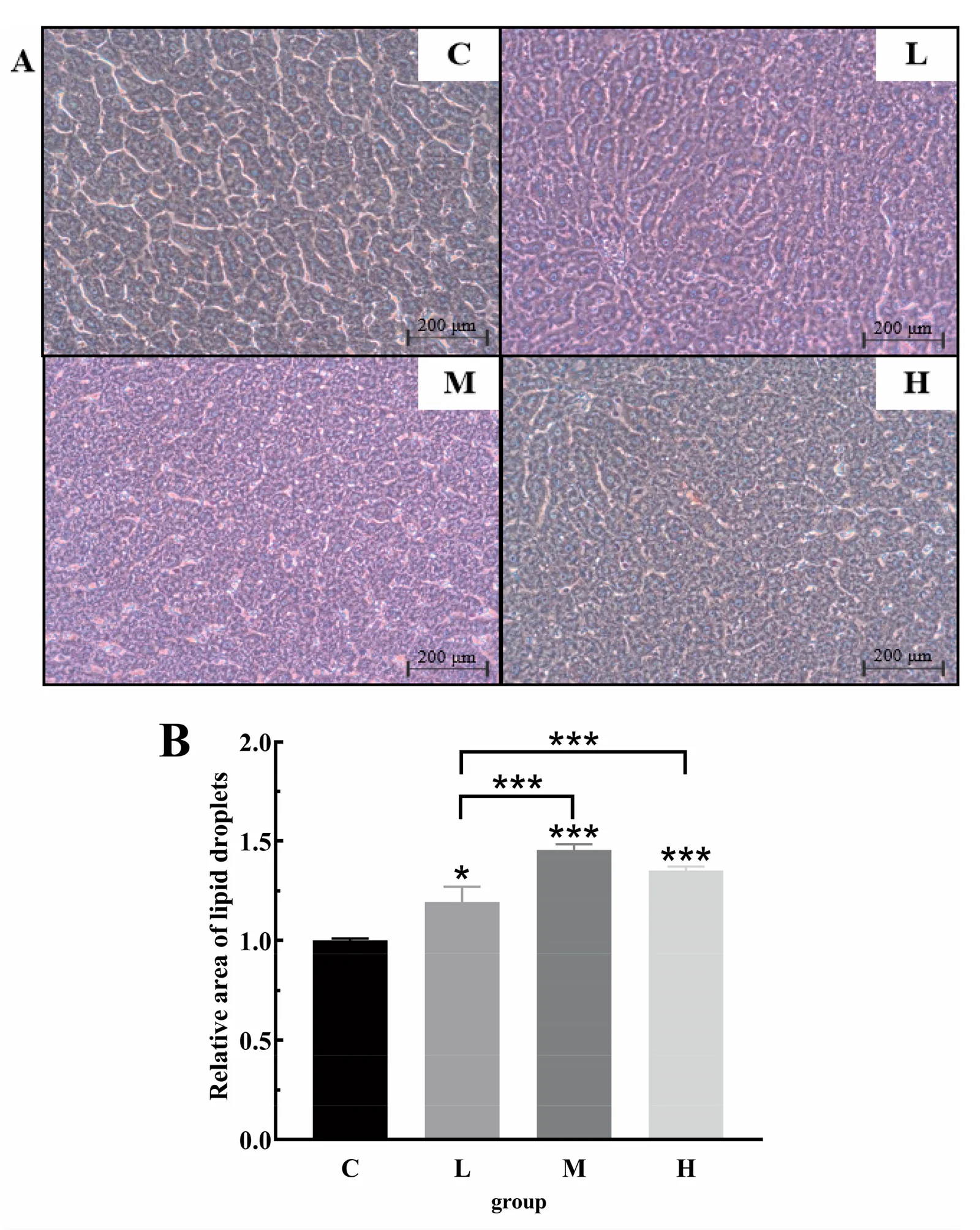

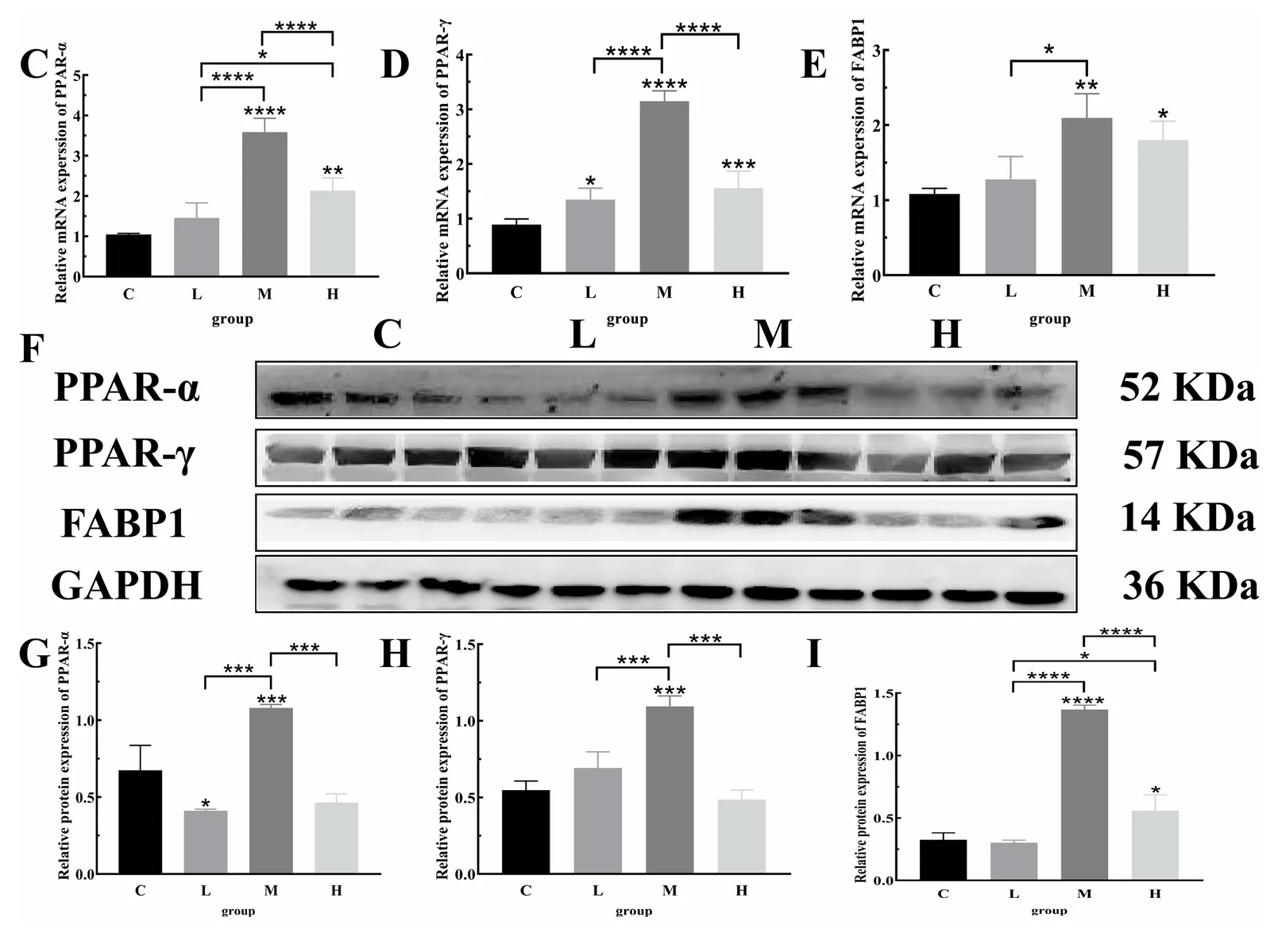
Effects of combined Flu/Phe exposure on hepatic lipid metabolism. (**A**) Oil Red O staining of liver (100×). (**B**) Lipid droplet area quantified by ImageJ analysis of Oil Red O-stained sections. (**C**) PPAR-α mRNA. (**D**) PPAR-γ mRNA. (**E**) FABP1 mRNA. (**F**) Western blot bands. (**G**) PPAR-α protein (semi-quantitative). (**H**) PPAR-γ protein (semi-quantitative). (**I**) FABP1 protein (semi-quantitative). Data are presented as mean ± SD (*n* = 5 rats per group). Statistical significance was determined by one-way ANOVA followed by Tukey’s test. * ***p*** < 0.05, ** ***p*** < 0.01, *** ***p*** < 0.001, **** ***p*** < 0.0001 compared with the C group. All analyses were performed blinded to group allocation. C, control; L, low dose; M, medium dose; H, high dose.

**Figure 13:**
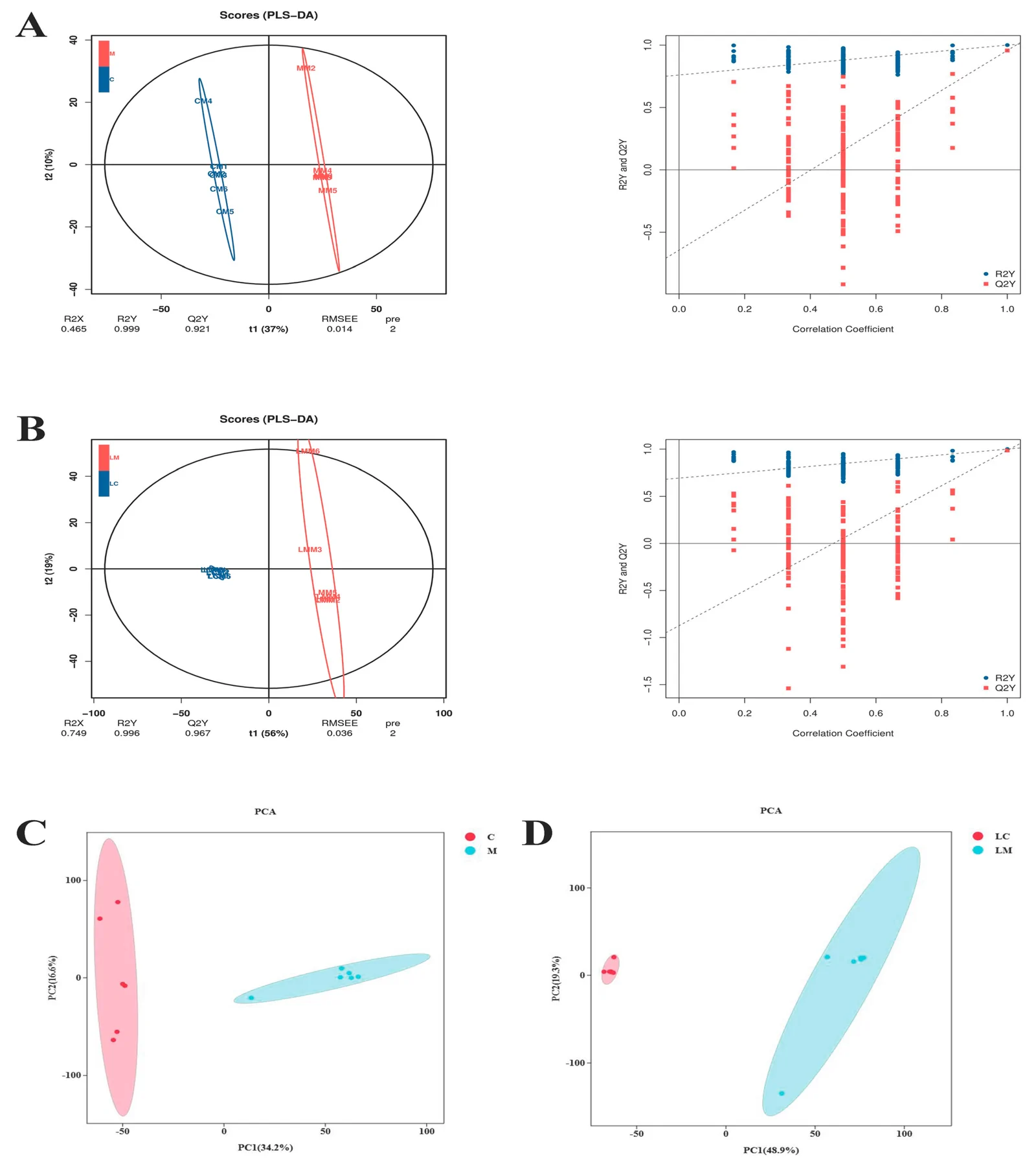

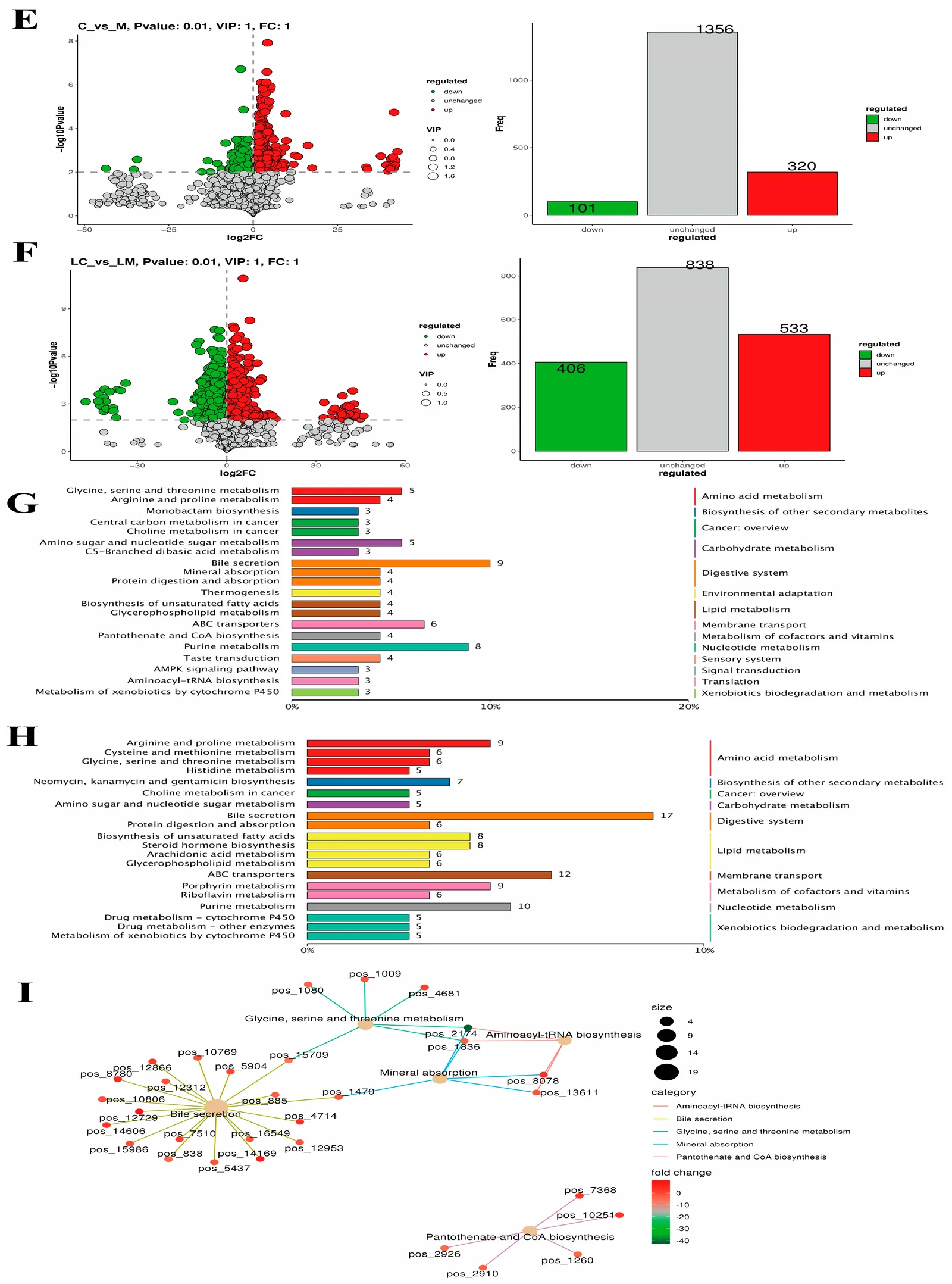
Metabolomics analyses of the gut–liver axis. (**A**) PLS-DA and permutation test (anion mode) for colon contents in C and M groups. (**B**) PLS-DA and permutation test (anion mode) for liver in C and M groups. (**C**) PCA of colon contents in C and M groups. (**D**) PCA of liver in C and M groups. (**E**) Differential metabolite analysis of colon contents. (**F**) Differential metabolite analysis of liver. (**G**) KEGG pathway analysis of colon contents. (**H**) KEGG pathway analysis of liver. (**I**) Differential metabolite KEGG enrichment network map. Data are presented as mean ± SD (*n* = 5 rats per group). Differential metabolites were identified using Student’s *t*-test ***p*** < 0.01, VIP > 1, and fold-change > 1 (403 metabolites in colon contents: 101 decreased, 302 increased; 939 in the liver: 406 decreased, 533 increased). Benjamini–Hochberg false-discovery-rate correction was applied, and the differential metabolites remained significant at FDR-adjusted ***p*** < 0.05. Hypergeometric test was used for KEGG enrichment. Permutation tests (200 iterations) validated the PLS-DA models. All analyses were performed blinded to group allocation. C, control; M, medium dose.

**Figure 14:**
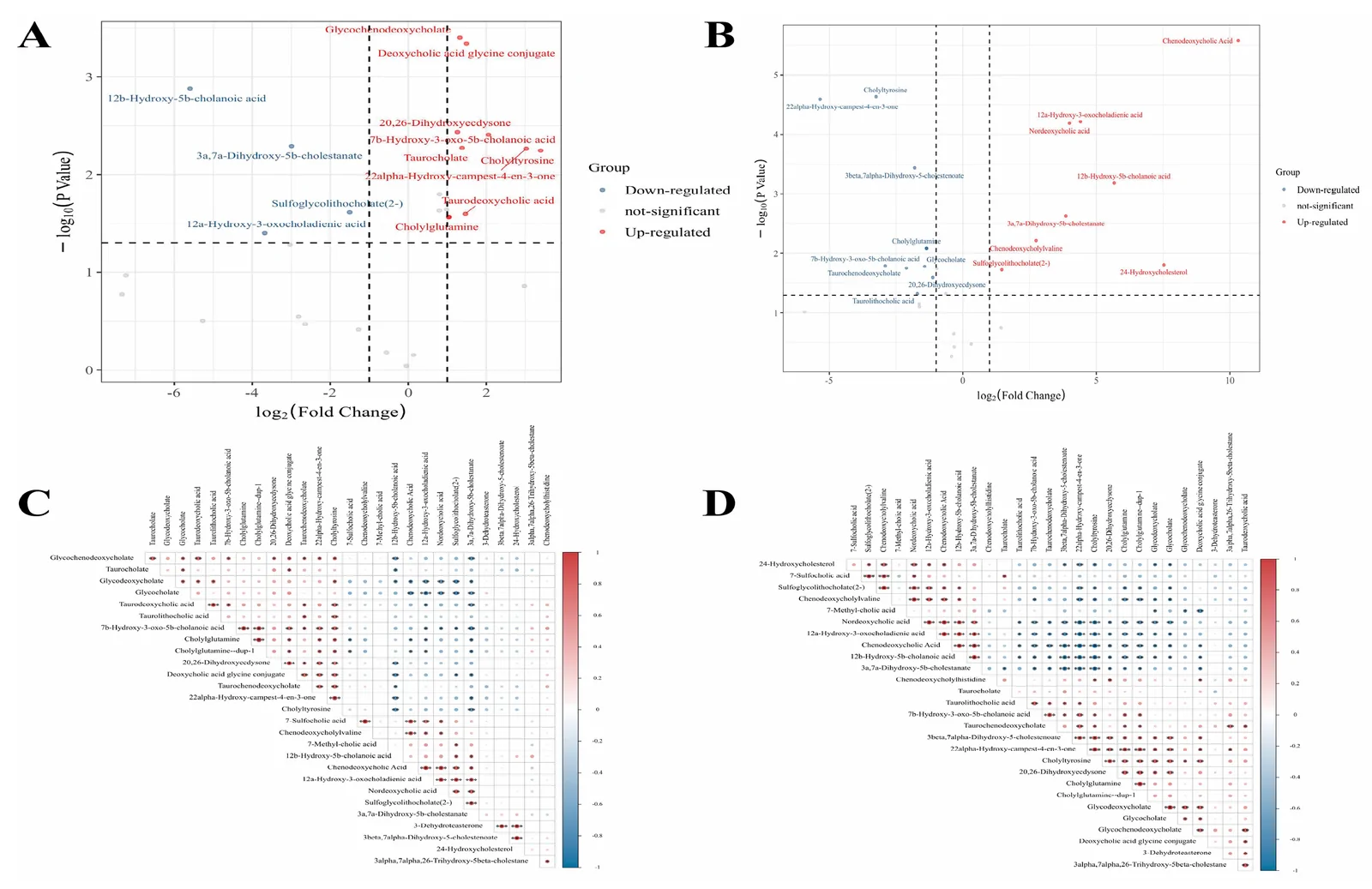

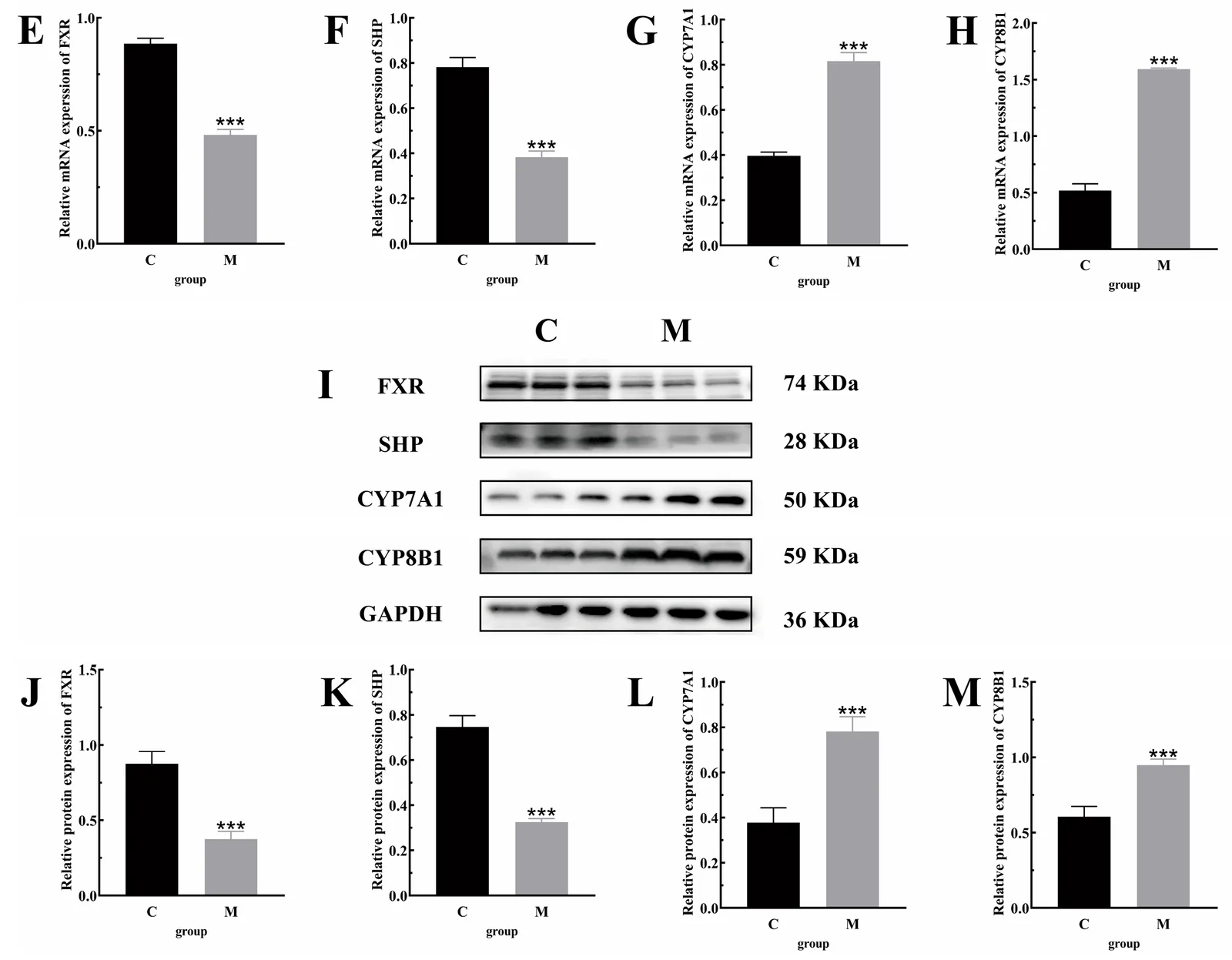
Analysis of bile acid metabolism in the gut–liver axis. (**A**) Volcano plot of bile acids in colon contents (C vs. M). (**B**) Volcano plot of bile acids in liver (C vs. M). (**C**) Bile acid correlation heat map for colon contents. (**D**) Bile acid correlation heat map for liver. For metabolomics analyses (**A**–**D**), *n* = 5 rats per group. (**E**) FXR mRNA. (**F**) SHP mRNA. (**G**) CYP7A1 mRNA. (**H**) CYP8B1 mRNA. (**I**) Western blot bands. (**J**) FXR protein (semi-quantitative). (**K**) SHP protein (semi-quantitative). (**L**) CYP7A1 protein (semi-quantitative). (**M**) CYP8B1 protein (semi-quantitative). For RT-qPCR and Western blot analyses (**E**–**M**), data are presented as mean ± SD (*n* = 5 rats per group). * ***p*** < 0.05, ** ***p*** < 0.01, *** ***p*** < 0.001, compared with the C group. All analyses were performed blinded to group allocation. C, control; M, medium dose.

**Figure 15:**
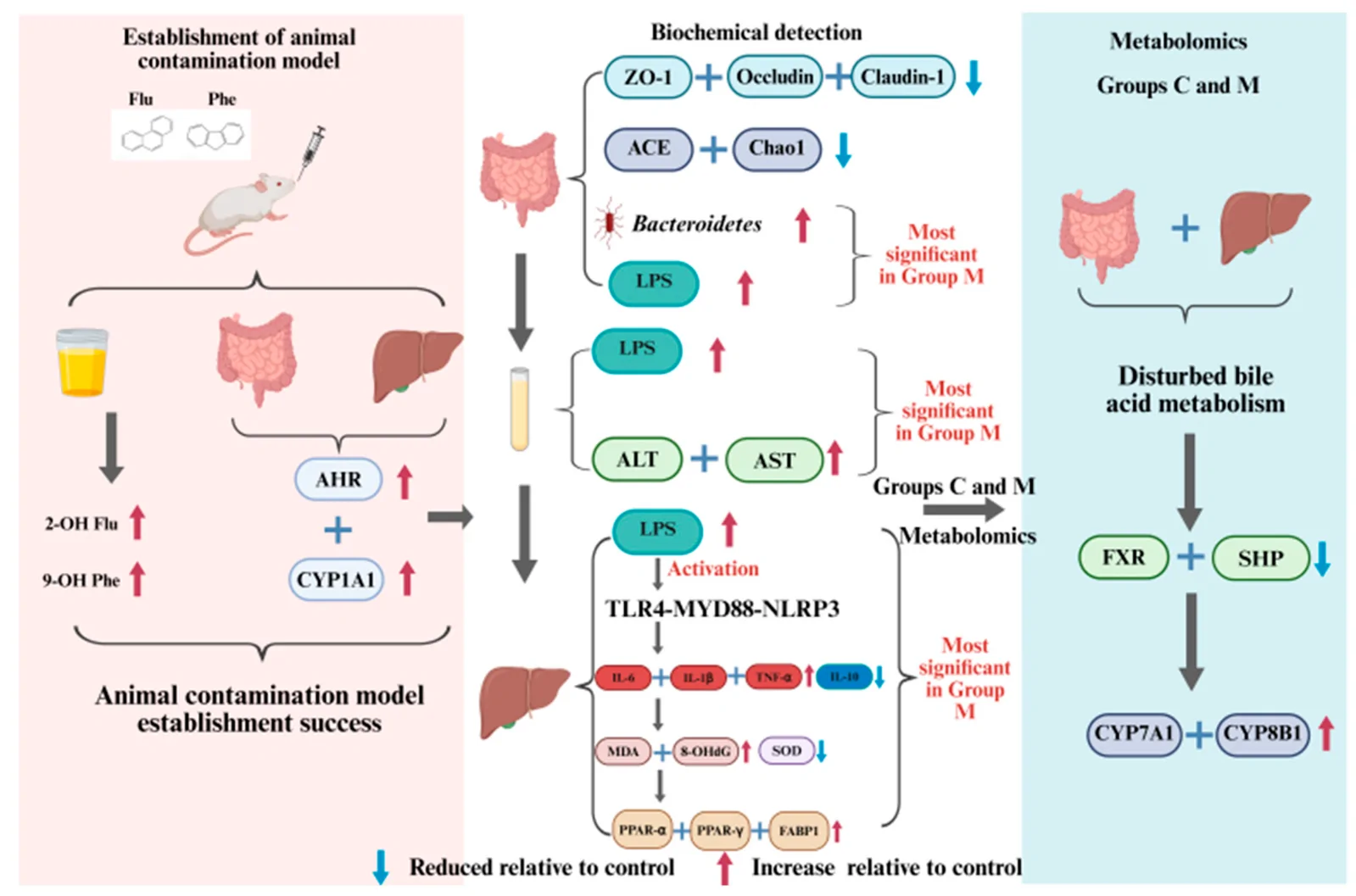
LMW-PAHs Induce Liver Injury via the Gut–Liver Axis: Identification of the Optimal Intervention Dose and Key Molecular Targets.

## Data Availability

The original contributions presented in this study are included in the article/Supplementary Materials. Further inquiries can be directed to the corresponding authors.

## Supplementary Materials

The following supporting information can be downloaded at: https://www.mdpi.com/article/10.3390/foods15193387/s1, Figure S1: Results of hepatic apoptosis; Tables S1 and S2: LEfSe data information and OH-PAH data results; Table S3: Statistical details; Table S4: Linear-trend and nonlinear (deviation-from-linearity) analysis of dose–response relationships.

## Appendix A. Material and Methods

### Appendix A.1. Detection of 9-OH Phe and 2-OH Flu in Urine

Hydrolysis enzyme solution: Weigh 60 mg of β-glucosidase (Type B-1, ≥1,000,000 units/g, McLean, Shanghai, China) and 140 mg of aryl sulfatase (Type H-1, ≥10,000 units/g, Merck, Darmstadt, Germany), add acetic acid-sodium acetate buffer solution, dissolve, and create up to 100 mL. A blend of 600 U/mL β-glucosidase and 14 U/mL arylsulfatase was synthesized as a ready-to-use hydrolytic enzyme solution.

The rat urine was mixed thoroughly, and 5.0 mL was transferred to a 25 mL centrifuge tube. 5.0 mL of mixed hydrolase solution was then added, and the mixture was hydrolyzed overnight at 37 °C in a thermostatic water bath, protected from light. After hydrolysis, the urine sample was centrifuged at 5000 rpm for 5 min, and 5.0 mL of the supernatant was transferred into a 10 mL stoppered cuvette. 4.0 mL of methanol was then added, and the supernatant was diluted with purified water. The supernatant was filtered through a 0.45 μm membrane and then determined on a PC.

### Appendix A.2. Gut Microbiota Analysis

#### Appendix A.2.1. DNA Extraction and Sequencing

DNA from fecal samples was extracted using the E.Z.N.A™ Mag-Bind Soil DNA Kit (Omega Biotech, Norcross, GA, USA). The V3-V4 variable region of the 16S rRNA gene was amplified using primers 338F (5′-ACTCCTACGGGAGGCAGCA-3′) and 806R (5′-GGACTACHVGGGTWTCTAAT-3′). The amplified products were sequenced on the Illumina MiSeq platform (PE250, Illumina, San Diego, CA, USA) to generate raw sequence data.

#### Appendix A.2.2. Data Processing

Raw reads were quality-filtered using Trimmomatic v0.33, and primer sequences were removed using cutadapt 1.9.1. Denoising, paired-end merging, and chimera removal were performed using the DADA2 method in QIIME2. The resulting non-chimeric reads were clustered into operational taxonomic units (OTUs) using Vsearch software (v2.31.0).

#### Appendix A.2.3. Taxonomic Assignment

Species classification and annotation were performed using the SILVA database (version 138) as the reference, with a Naive Bayes classifier at a confidence threshold of 0.7.

#### Appendix A.2.4. Statistical and Bioinformatics Analyses

α-diversity (ACE, Chao1, Simpson, Shannon), β-diversity (PCoA based on Bray-Curtis distances), species difference analysis, marker species screening, and functional prediction were performed using the online platform of Biomarker Technologies Co., Ltd. (URL: https://international.biocloud.net/). LEfSe (Linear Discriminant Analysis Effect Size) was performed with an LDA score threshold > 3.0 to identify differentially abundant taxa among groups.

### Appendix A.3. RT-qPCR Analysis

The primers are presented in Supplementary Table S1. PCR procedure: pre-denaturation at 95 °C for 30 s, followed by 40 cycles (95 °C for 5 s, 55–65 °C for 30 s), and a final extension at 95 °C for 15 s. The expression level of the target genes was normalised to the expression level of the Gapdh gene by the 2-DDCt technique. Gapdh gene expression level following relative quantitative analysis.

**Table A1:** Primer sequences.

Gene	(5′-3′) Sequence
GAPDH	Forward: TATGACAACAGCCTCAAGAT Reverse: AGTCCTTCCACGATACCA
PPAR-α	Forward: ACGATGTGTCCTCCTTGATGAAC Reverse: ATGATGTCGCAGAATGGCTTCCTC
PPAR-γ	Forward: CCATCGAGGACATCCAAGACAA Reverse: GTGCTCTGTGACAATCTGCCTGA
FABP1	Forward: AGCCAAGAGAACTTTGAGCCT Reverse: TGGTCTCCAGTTCGCACTCCTC
IL-6	Forward: ACTTCCAGCCAGTTGCCTTCTT Reverse: TGGTCTGTTGTGGGTGGTATCC
IL-1β	Forward: GACAAGAGCTTCAGGAAGGCA Reverse: TCATCATCCCACGAGTCACAG
TNF-α	Forward: ATGGGCTCCCTCTCATCAGTT Reverse: GCTCCTCCGCTTGGTGGTTTG
IL-10	Forward: GGTTGCCAAGCCTTGTCAGAA Reverse: CTTCACCTGCTCCACTGCCTTG
TLR4	Forward: TGTATCGGTGGTCAGTGTGC Reverse: CAGCTCGTTTCTCACCCAGT
MyD88	Forward: GAGAGCAGTGTCCCACAGAC Reverse: AGCAGATGAAGGCGTCGAAA
NLRP3	Forward: CCAGGAGTTCTTTGCTTTGCGGCTA Reverse: GCCTTATTCGAACTTGCCGT
ZO-1	Forward: GCCTCCTCCTCCTCCGTTGC Reverse: AGTTGCTGGTTTGCTCTGAG
Occludin	Forward: CAACGGCAAAGTGAATGGCA Reverse: TCATCCACGGACAAGGTCAGA
Claudin-1	Forward: CCTGGCTTCTCTGGGATGGATC Reverse: CTGAGCGGTCACGATGTTGTC
CYP1A1	Forward: TGCCAATGTCCAGCTCTCAG Reverse: TCCCTGCCAATCACTGTGTC
AHR	Forward: AGAGTTAGACCGCCTGGCTA Reverse: GTCAGCCGGTGTGGATTGTA
FXR	Forward: CCTCCTCGTCCTACTATTCCAACC Reverse: GCATTCGCCTGAGTTCATAGAGTC
SHP	Forward: TCCCGTGTCTTTCAGCTGTC Reverse: TAGCAGTGACTGGTTGCAGG
CYP7A1	Forward: GAATAGCTGGCTGAGGAAT Reverse: TCAAAGGTGGAGAGCGTGTC
CYP8B1	Forward: GCCGACGACCTTCAAGTCA Reverse: CTGCCGGGACATATGGAGAC

### Appendix A.4. Western Blotting

Proteins were extracted by lysing colon and liver tissues using RIPA lysis buffer (PhD Wuhan, China) containing 1% PMSF (PhD Wuhan, China) and 1% phosphatase inhibitor (PhD Wuhan, China). The entire protein concentration was subsequently measured using the BCA assay (PhD Wuhan, China). The lysates were separated by sodium dodecyl sulfate-polyacrylamide gel electrophoresis (SDS-PAGE) (Bio-Rad, Hercules, CA, USA). After electrophoresis, proteins were transferred to polyvinylidene difluoride (PVDF) membranes. The membrane was closed with ice bath free rapid closure solution (Shanghai He Yuan Li Ji, Shanghai, China) for 15 min at room temperature, and then with GAPDH, PPAR-α, PPAR-γ, FABP1, IL-1β, IL-6, IL-10, TNF-α, TLR4, MyD88, NLRP3, ZO-1, Occludin, Claudin-1, CYP1A1, AHR, FXR, SHP, CYP7A1, and CYP8B1 antibodies were incubated at 4 °C for 12-14 h. The protein levels were then analyzed using an anti-rabbit IgG secondary antibody for 1 h at room temperature, utilizing a gel imaging system (Universal Hood II, U.S.A. Bio-Rad).

**Table A2:** Antibodies used for western blotting.

Antibodies	Dilution	Description
GAPDH	1:1000	SAB, 52902
PPAR-α	1:1000	SAB, 41359
PPAR-γ	1:1000	SAB, 41360
FABP1	1:1000	SAB, 32764
IL-1β	1:1000	SAB, 41059
IL-6	1:1000	SAB, 41739
IL-10	1:1000	SAB, 41553
TNF-α	1:1000	SAB, 41504
TLR4	1:1000	SAB, 35463
MyD88	1:1000	SAB, 32107
NLRP3	1:1000	SAB, 49012
ZO-1	1:1000	SAB, 29274
Occludin	1:1000	SAB, 29275
Claudin-1	1:1000	SAB, 40747
CYP1A1	1:1000	SAB, 32629
AHR	1:1000	SAB, 53865
FXR	1:1000	SAB, 56795
SHP	1:1000	BIoss, bs-4311R
CYP7A1	1:1000	SAB, 29556
CYP8B1	1:1000	Cusabio, Q9UNU6
Goat Anti-Rabbit IgG	1:10,000	SAB, L35017
