# Supplementary material for: The Gut–Liver Axis Mediates Liver Injury Induced by Low-Molecular-Weight Polycyclic Aromatic Hydrocarbons Through TLR4-Dependent Inflammation and Bile Acid Dysregulation
Source: Foods. 2026 Sep 23;15(19):3387. doi: 10.3390/foods15193387 (PMC13664197; doi:10.3390/foods15193387)
Supplement: Supplementary file 1 [file foods-15-03387-s001.zip › Fig. S1.pdf]

Results of hepatocyte apoptosis

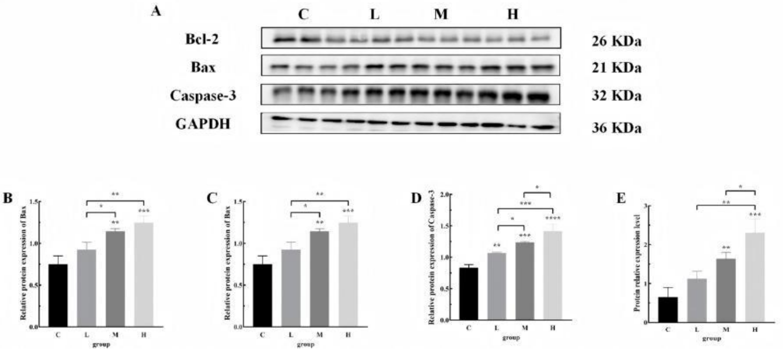

Fig. S1 Expression levels of apoptosis-related proteins. A is the Western blot protein band; B is the protein band of Bcl-2 bands; C is the protein band of Bax bands; D is the protein band of Caspase bands; E is relative protein expression ratio of Bax/Bcl-2. n=3 per group, three independent replicates.

Compared with the control and M groups, the H group exhibited a significant increase in the Bax/Bcl-2 ratio (***P*** < 0.05) and elevated cleaved Caspase-3 expression (***P*** < 0.05), indicating activation of the mitochondrial apoptotic pathway at the high dose.
